# Hybrid nanocellulose material as an adsorbent to remove reactive yellow 2 dye

**DOI:** 10.1038/s41598-024-70906-5

**Published:** 2024-08-29

**Authors:** Beatris L. Mello, Pascal S. Thue, Pâmela V. da Silva, Caroline Saucier, Glaydson S. dos Reis, Fernando M. Machado, Rafael de Avila Delucis, Mu. Naushad, Farooq Sher, Moaaz K. Seliem, Eder C. Lima

**Affiliations:** 1 Institute of Chemistry, Federal University of Rio Grande do Sul (UFRGS), Bento Gonçalves 9500, Porto Alegre, RS Brazil; 2 Environmental Science Graduate Program, Engineering Center, Federal University of Pelotas (UFPel), 989 Benjamin Constant St., Pelotas, RS 96010-020 Brazil; 3 Department of Forest Biomaterials and Technology, Biomass Technology Centre, Swedish University of Agricultural Sciences, 90183 Umeå, Sweden; 4 Technology Development Center, Federal University of Pelotas (UFPel), 1 Gomes Carneiro St., Pelotas, RS 96010-610 Brazil; 5 Department of Chemistry, College of Science, King Saud University, P.O. Box 2455, Riyadh, 11451 Saudi Arabia; 6 Department of Engineering, School of Science and Technology, Nottingham Trent University, Nottingham, NG11 8NS UK; 7 Faculty of Earth Science, Beni-Suef University, Beni Suef, 62511 Egypt

**Keywords:** Wastewater treatment, Adsorption, Reactive yellow 2 textile dye, Sustainable development goals, Nonlinear van’t Hoff equation, Chemistry, Materials science

## Abstract

Textile dyes are frequently disposable in aqueous effluents, making it difficult to remove them from industrial effluents before their release to natural waters. This paper deals with the fabrication of cellulose-based adsorbents by reacting nanocelulose crystalline (nanocel) with *N*-[3-(trimethoxysilyl)propyl]ethylenediamine (TMSPEDA), forming the hybrid (silylpropyl)ethylenediamine@nanocellulose (SPEDA@nanocel), which was employed as adsorbent for the uptake of reactive yellow 2 dye (RY-2) from aqueous effluents. Characterisation of SPEDA@nanocel was carried out using FTIR, SEM–EDS, XRD, TGA, surface area, pH_pzc_, and hydrophobicity/hydrophilicity ratio (HI). Also, adsorption studies were thoroughly investigated. The effect of initial pH indicated that the maximum uptake of RY-2 takes place at pH 2, which is an indication of the electrostatic mechanism. The kinetic data carried out with 250 and 500 mg L^−1^ RY-2 with SPEDA@nanocel followed better the nonlinear fractional-like pseudo-first-order model. The t_0.5_ and t_0.95_ for the dye uptake were about 30 and 141 min, respectively. The equilibrium data from 10 to 45 °C indicated that the Liu isotherm model was the best-fitted isothermal model. The maximum sorption capacity attained was 112.3 mg g^−1^ at 45 °C. The thermodynamic data have shown that the equilibrium was favorable and endothermic, and the ΔH° was compatible with an electrostatic attraction between RY-2 and SPEDA@nanocel. Experiments of desorption of loaded adsorbent showed promising results for real applications since at least 5 adsorption/desorption cycles could be employed without significant changes in the recovery and with high precision.

## Introduction

In 2022, almost 2 billion persons did not have access to potable drinking water^1^. In the water pollution environmental matter, dyes are one of the most found organic pollutants present in industrial streams, getting worse in the water crisis worldwide^1–3^. Dyes are usually employed in different industrial sectors, such as printing ink, food, drugs, and fabrics^4^. Dyes present a rigid molecular structure, becoming refractory, making their degradation difficult and augmenting their persistence in the environment^5^. Dyes present in waters can deteriorate aquatic life by decreasing the dissolved oxygen concentration, augmenting the biological and chemical oxygen demand, changing the pH of aquatic systems, and altering the absorption of light, impacting the photosynthesis of aquatic plants^5,6^. Dyes are toxic and refractory, making them bioaccumulating, mutagenic, and carcinogenic^5–9^. Therefore, wastewater containing dyes should be treated before being released into the environment. Reactive Yellow 2 dye (RY-2; C.I.18972; CAS 50662-99-2)^10^ is primarily used for dying textiles. RY-2 reactive dye is commercialized with at least 39 trade names^10^. Therefore, removing this dye from dye effluents before the wastewater is disposed of in water bodies is an environmental concern because, according to EPA (US Environmental Protection Agency), the amount of reactive dyes that could be released into the wastewater varies from 50 to 60%^5^, and following OECD (Organization for Economic Cooperation Development), the limit ranges from 20 to 50%^5^. Concerning 1–2 mg L^−1^ of a dye being able to color a water body, it is crucial to have treatment methods that produce water with contents of dyes < 1 mg L^−1^^5^.

A paramount number of treatment methods are usually utilized for dye removal from aqueous wastewater. The most usual methods are biodegradation^11,12^, nanofiltration^13,14^, Fenton-like oxidation^15,16^, electrochemical degradation^17,18^, catalysis^19,20^, photocatalysis^21,22^, ultrasound degradation^23,24^, microwave degradation^25,26^ and adsorption^27–36^. Although all these methods are applicable, adsorption stands out over the other methods for dye removal because these methods are expensive^13,17,19–26^, being prone to the formation of transformation products with toxicity that could surpass the original molecule^15–26^, besides the generation of sludges^15–26^. Adsorption is an adequate treatment method for removing dyes from the environment because of its apparatus simplicity, which leads to low investment costs and high efficiency and allows the regeneration of loaded adsorbent by applying different adsorbent materials^37–40^. Among these adsorbents frequently employed for the removal of pollutants, activated carbon is the most employed because of the advantageous carbon surface that leads to high sorption capacities^41–43^. On the other hand, carbon-activated fabrication presents some drawbacks, like high energy consumption in biomass pyrolysis and low recovery rates for regeneration of used adsorbent^37,38^. Nevertheless, cellulose-based adsorbents are attractive due to their low cost, high abundance, low toxicity, and high sorption capacities^3,31,44^. Cellulose is extracted from wood, cotton, and plants. Cellulose fibers can be used for several ends, such as fertilizer^45^, packaging and papermaking, and the uptake of pollutants^3,31,44^. It is well known that pure biomass materials such as cellulose are not promising adsorbents for the uptake of pollutants; therefore, chemically modified cellulose-based materials present better adsorption characteristics^3,31,44,46^. Multiporous ZIF-8 carbon/cellulose nanocomposite beads were synthesized and employed to uptake Rhodamine B (RhB) dye from aqueous effluents. The metal–organic framework and cellulose composite material presented an elevated surface area (> 1400 m^2^ g^−1^). The maximum sorption capacity based on the Langmuir isotherm model attained a value of 565.13 mg g^−1^. The adsorbent presented an excellent recovery of up to 5 cycles of adsorption/desorption^46^. An eco-friendly composite adsorbent prepared from kaolin clay and cellulose extracted from peanut shells to remove methylene blue (MB) and congo red (CR) dyes was proposed^47^. The synthesis of the composite adsorbent was optimized using the Box-Behnken design. The surface area of the composite material ranged from 39 to 96 m^2^ g^−1^. Based on the Langmuir isotherm models, the maximum sorption capacities attained were 291.5 mg g^−1^ (MB) and 130.7 mg g^−1^ (CR)^47^.

Besides these outstanding adsorbents utilized for dye adsorption^3,32–34,40,42–44,46^, adsorbent materials used to remove organic molecules can be fabricated from silylant reactants, like 3-aminopropyl-triethoxysilane (APTES), *N*^1^-(3-Trimethoxysilyl-propyl)-Diethylenetriamine (TMSPDETA). APTES and TMSPDETA add nucleophile moieties to solid supports, facilitating dye uptake^27–31,47–49^. The grafting of organosilane groups to cellulose-based materials is an effective method for creating adsorbent materials with a higher dye adsorption capacity compared to untreated biomass^27–31,47–49^. The development of new cellulose-based materials that have been chemically modified with organosilane groups is crucial. This paper focuses on grafting N-[3-(trimethoxysilyl)propyl]ethylenediamine (TMSPEDA) onto the surface of nanocellulose crystalline to enhance Reactive Yellow 2 (RY-2) dye uptake from aqueous effluents. The nano cellulose crystalline (nanocell) and hybrid material (SPEDA@nanocel) underwent characterization using scanning electron microscopy (SEM), energy dispersive x-ray spectroscopy (EDS), Fourier-transform infrared spectroscopy (FTIR), pH at the point of zero charge (pH_pzc_), x-ray powder diffraction (XRD), hydrophobicity-hydrophilicity ratio (HI), thermal gravimetric analysis (TGA), and N_2_-adsorption/desorption analysis. The adsorption data was adjusted using nonlinear kinetic, isotherm, and thermodynamic models. Additionally, the used adsorbent was regenerated, allowing for its reuse after 5 cycles of adsorption/desorption without significant changes in the recoveries and with elevated precision. This process maintained a high sorption capacity, making the use of SPEDA@nanocel cost-effective for the uptake of RY-2 dye.

## Materials and methods

### Chemicals

Dye solutions were produced with distilled water (Permution). Reactive Yellow 2 (RY-2), also commercially available as Cibacron Brilliant Yellow 3G-P, was provided by Sigma-Aldrich and used as received (see Fig S1). Solutions of 0.1 M HCl and NaOH were utilized for pH adjustments. CelluForce Industry, Montreal, Canada, provided nanocrystalline cellulose (nanocel). This material was utilized to prepare the hybrid SPEDA@nanocel. N-[3-(trimethoxysilyl)propyl]ethylenediamine (TMSPEDA) was provided by Sigma-Aldrich and used as a grafting species for the fabrication of SPEDA@nanocel.

### Grafting of TMSPEDA on nanocel

**Fig. 1:**
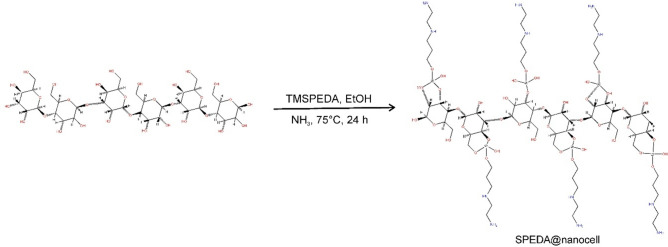
Scheme of grafting of SPEDA on nanocellulose.

The chemical modification of nanocrystalline cellulose with TMSPEDA for producing the SPEDA@nanocel hybrid material was carried out by employing NH_4_OH (28%) to catalyze the hydrolysis of alkoxy groups of TMSPEDA in ethanol. An amount of 10.00 g of nanocel was slurried with 100.0 mL of ethanol, and 100 µL of NH_3_ was added^27,31^. Subsequently, 2.50 g of TMSPEDA (99%) was added to the mixture. The final pH of the mixture was 10–11. Further, the reactional mixture was magnetically stirred for 24 h at 70–75 °C under a continuous reflux device. Further, the brown slurry was filtered under pressure and rinsed thoroughly with water: ethanol 1 + 1, followed by distilled water to withdraw the unreacted TMSPEDA from the final product. Afterward, the filtered adsorbent was dried in a conventional oven at 85 °C for 30 h. The modified nanocrystalline cellulose was defined as SPEDA@nanocel, while the pure nanocellulose was denoted (nanocel). The diagrammatic scheme of the grafting of TMSPEDA onto cellulose is described in Fig. 1.

### Characterization

The nanocellulose (nanocel) and the SPEDA@nanocle materials were characterized by different analytical techniques for characterization, such as SEM, EDS^50^, FTIR^51^, TGA^52^, X-ray-Diffraction^53^, pH_pzc_^54^, and hydrophobic properties (HI)^55^, and surface area^56^.

### Adsorption experiments

Adsorption experiments of removal of RY-2 dye onto SPEDA@nanocel were carried out at 10–45 °C, using 20.00 mL dye solution and 30.0 mg adsorbent, at pH ranging from 2.0 to 8.0^39,54,55^. Analytical control concerned with the determination of RY-2 is given in detail in Supplemental File^54^. Adsorption kinetics, equilibrium, and thermodynamics were employed using nonlinear fitting^57–59^. The statistical analysis of the adsorption models was performed based on values of R^2^_adj_, standard deviation (SD), and BIC values^57,60^. See the Supplementary material^39,54,55,57–60^.

## Results and discussion

### SEM and EDS

**Fig. 2:**
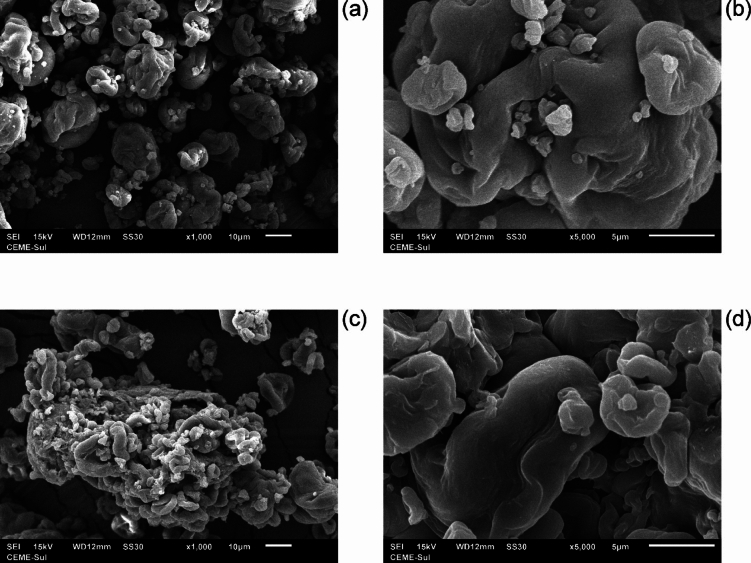
SEM images (**a**) nanocell at 1000X magnification (**b**) nanocell at 5000X magnification, (**c**) SPEDA@nanocel 1000X magnification, (**d**) SPEDA@nanocel 5000X magnification.

The morphological analysis of modified nanocrystalline cellulose (SPEDA@nanocel) and nanocellulose (nanocel) materials is achieved using SEM equipment. The images are taken at 1000× and 5000× magnifications and are shown in Fig. 2. Figure 2a and b present the images of nanocrystalline cellulose without any modification. It is clear from the images to observe an irregular shape of the particles, added to a smooth surface, and compact spheric-like aggregates. Compared to the cellulose microcrystalline, which shows compact rodlike aggregates^31^, nanocrystalline cellulose shows spheric-like aggregates. Therefore, using different particle sizes of the cellulose in the grafting process may also lead to different materials. Figure 2c and d present the modified nanocrystalline cellulose (SPEDA@nanocel) images—these images are slightly different from the nanocel. SPEDA@nanocel shows the roughest and smoothest surface of the material compared to the nanocel images. More particles at the surface were observed, probably attributed to the grafting of the TMSPEDA groups grafted to the nanocel. Such amino silane at the surface enhances the adsorption of Reactive Yellow 2 (RY-2) from an aqueous solution, which is likely to obtain efficient materials.

**Fig. 3:**
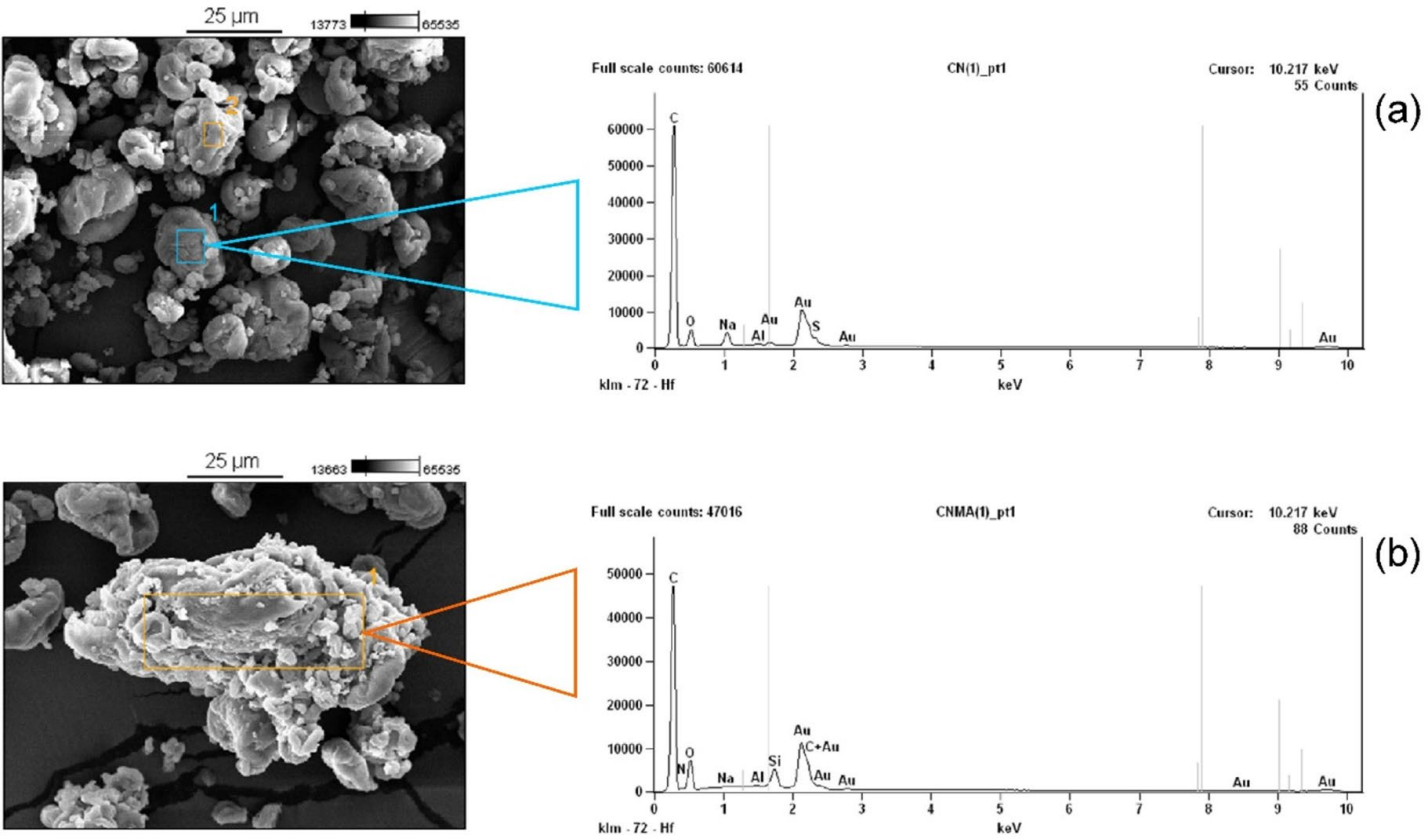
SEM–EDS images. (**a**) nanocell; (**b**) SPEDA@nanocell.

Energy-dispersive X-ray spectroscopy is a technique that provides the chemical composition of the material. This study uses that purpose to determine the chemical element in the modified and pure nanocellulose. Both data are critical to stating whether the grafting process is effective. The EDS result in Fig. 3 depicts the common element found in the cellulose. The main chemical elements are carbon and oxygen elements on both materials, belonging to the chemical structure of cellulose. It is interesting to figure out that the carbon element was higher than the value found by the elemental analysis for both materials (see Table 1), while the oxygen element was smaller in both materials. The C-containing and O-containing were 54.16% and 12.85% for the nanocel, while SPEDA@nanocel contains C (44.31%) and O (20.06%)^50^.

**Table 1:** Surface properties of nanocel and SPEDA@nanocel hybrid material.

	HI	pH_pzc_	BET Surface Area (m^2^ g^−1^)	Ash (%)	Grafting (mmol g^−1^)
Nanocel	0.151	6.715	0.1	0.0001	–
SPEDA@nanocel	0.210	9.309	0.4	4.87	0.811

Notwithstanding, SPEDA@nanocel presents peaks related to Si and N elements, which are not present in nanocel structure, suggesting that SPEDA moieties were grafted effectively on nanocel. In addition to the peaks related to carbon and oxygen, the EDS shows Si (3.06%) and N (3.39%). This result proves that *N*-[3-(trimethoxysilyl)propyl]ethylenediamine was present in the nanocellulose skeleton^27,29,31,50^.

### FTIR

**Fig. 4:**
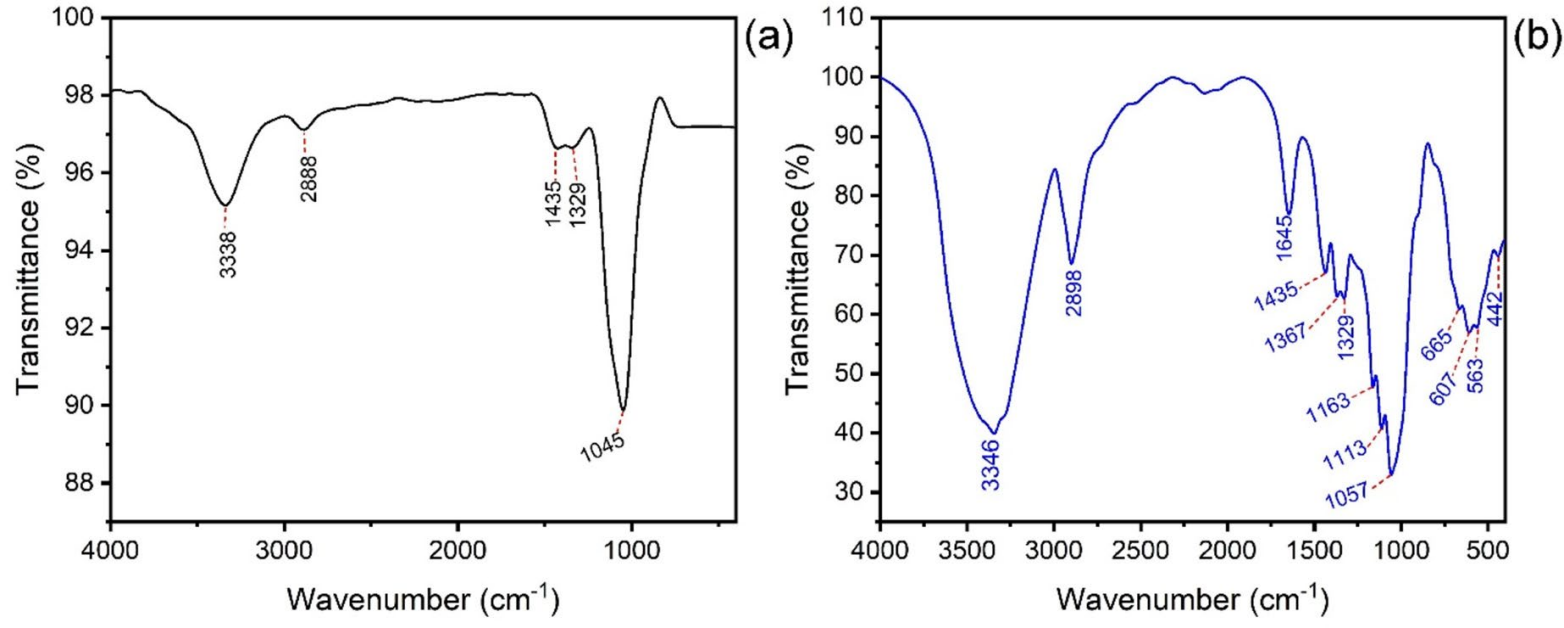
FTIR spectra of (**a**) nanocel and (**b**) SPEDA@nanocel.

The nanocel and SPEDA nanocell functional groups were evaluated using FTIR spectroscopy (4000–400 cm^−1^) (see Fig. 4). The broadbands centered at 3338 cm^−1^ (nanocel) and 3346 cm^−1^ are ascribed to O–H bonding stretching^27,30,51^. The bands at 2888 (nanocel) and 2898 cm^−1^ (SPEDA@nanocel) are ascribed to symmetric stretching C–H bond^31,41,51^. The band at 1645 cm^−1^ of SPEDA@nanocel is attributed to NH_2_ scissors^31,51^, which is absent in nanocell material. Both materials’ bands at 1435 cm^−1^ are ascribed to bends of C-CH_2_ groups^29,51^. The bands at 1329 (nanocel) and 1367 and 1329 cm^−1^ (SPEDA@nanocel) could be attributed to O–H bendings^51^. The band at 1045 cm^−1^ in nanocel is ascribed to the stretching of the C–O group. On the other hand, the hybrid material presents the following bands: 1163, 1113, and 1057.

cm^-1^ assigned to stretches of the C–O groups^28,51^ and asymmetric stretching of C–O–C groups^28,51^ that also could be confounded to asymmetric stretching of Si–O–Si^28,51^. The bands at 665 and 607 cm^−1^ are assigned to NH_2_ Wag bending^27,51^ presented in SPEDA@nanocel. The band at 442 cm^−1^ is attributed to Si–O–Si bending^30,51,55^. The prominent FTIR bands that differentiate the hybrid material from its precursor are 1645 cm^−1^ (bends scissors of NH_2_), 665, and 607.

cm^-1^ (bend of Wag NH_2_), indicating that SPEDA@nanocel was fabricated.

### Thermalgravimetric analysis

**Fig. 5:**
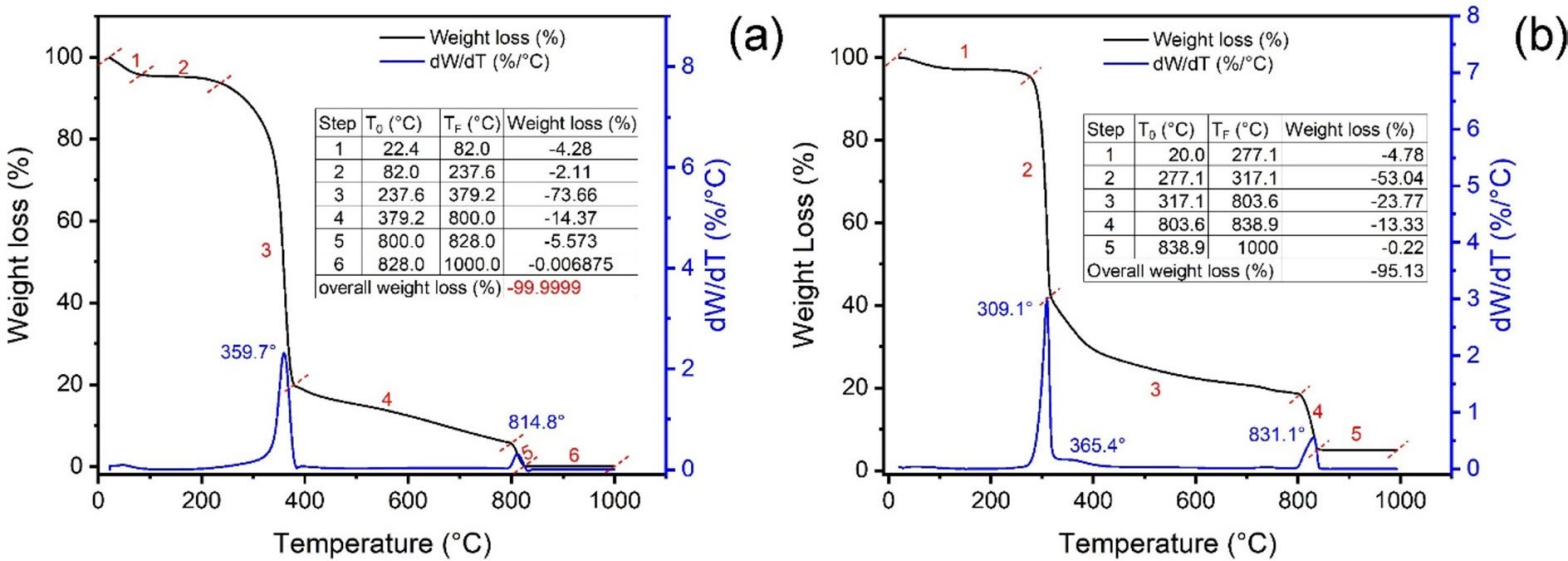
TGA and DTA of (**a**) nanocel; (**b**) SPEDA@nanocel.

The thermal behavior of nanocel and SPEDA@nanocel are depicted in Fig. 5. These experiments were carried out under N_2_ (20–800 °C) and synthetic air (800–1000 °C)^30,54^. The thermal profile of nanocel and SPEDA@nanocel have 6 and 5 weight loss steps, respectively. The first and 2nd steps of nanocel and 1st weight loss of hybrid material could be assigned to the weight of water (moisture and interstitial water)^30,54^, corresponding to 6.39% and 4.78% weight loss, respectively. The 3^rd^ step of weight loss of nanocel and 2nd step of weight loss of SPEDA@nanocel materials are assigned to the initial degradation of the cellulose skeleton, corresponding to 73.66 and 53.04% of weight loss^30,54^, which were the highest weight loss observed. The 4th (nanocel) and 3rd (hybrid material) weight loss steps took place from 379.2 to 800.0 °C (nanocel) and 317.1–803.6 °C (SPEDA@nanocel), corresponding to 14.37% (nanocel) and 13.33% (SPEDA@nanocel) weight losses. In the following steps, the atmosphere shifted from N_2_ to synthetic air, where all organic matter was degraded, generating ashes^30,54^. For nanocel, the ashes content is only 0.0001%, and for the hybrid material, it is 4.87%. Considering that all silicon content of SPEDA@nanocel was converted to SiO_2_, the total number of SPEDA groups attached to the nanocel can be estimated, whose value was 0.811 mmol g^−1^. Taking into account the amount of TMSPEDA used in the synthesis, it can be inferred that the yield of the chemical reaction depicted in Fig. 1 was 95.9%.

### XRD

**Fig. 6:**
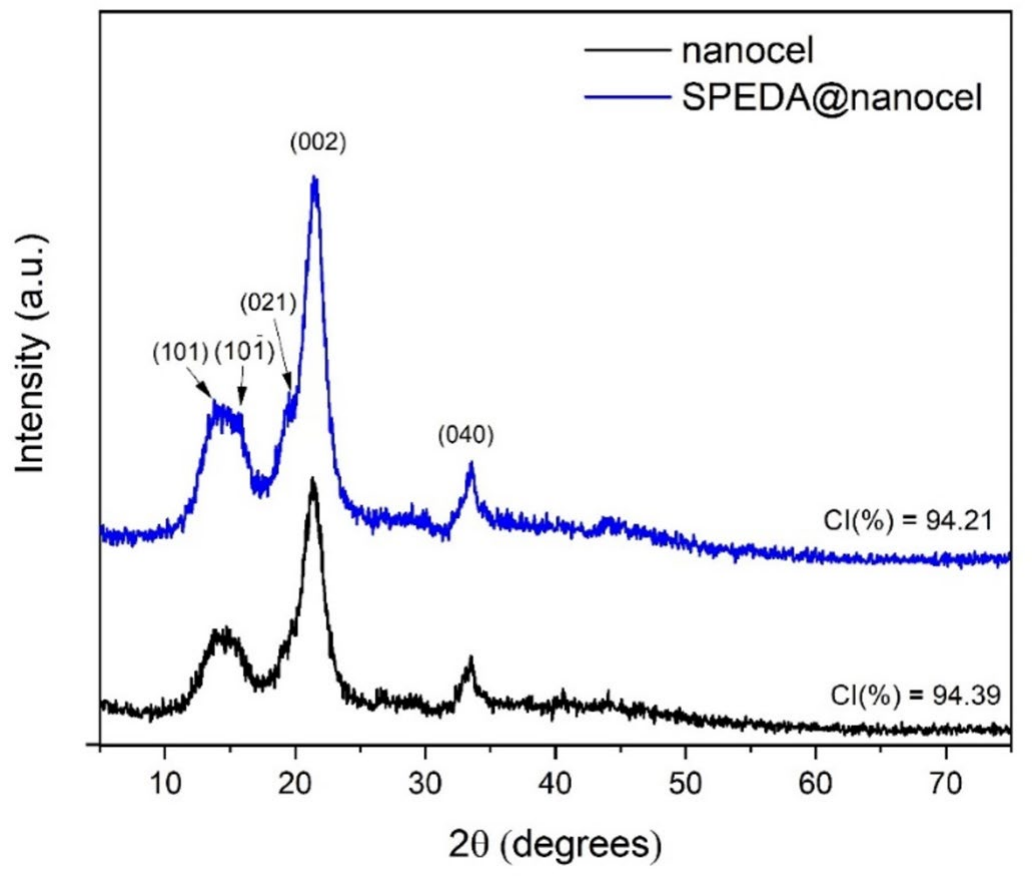
XRD patterns of nanocel and SPEDA@nanocel samples.

Figure 6 displays the X-ray diffraction patterns of the nanocel and SPEDA@nanocel adsorbent. Both diffractograms exhibit typical cellulose peaks at 13.9°, 15.9°, 19.7°, 21.2°, and 33.5°, corresponding to the (101), (10 $\overline{1 }$), (021), (200), and (040) reflections^31^. The crystallinity index (CI, calculated by Segal Method^53^) of the sample hybrid material is slightly lower (94.21%) compared to the CI of the nanocel sample (94.39%). Additionally, a slight rise in the amorphous scattering between the (10 $\overline{1 }$) and (002) peaks (~ 20 = 17–20°)^53^ is observed in the diffractogram of the grafted sample. These phenomena can be related to the increase in the amorphous fraction of the SPEDA@nanocel sample, resulting from the reduction of intra- and intermolecular hydrogen bonds in nanocrystalline cellulose due to the modification process with TMSPDETA^31^.

### pHpzc and HI, the BET surface area of SPEDA@nanocel

Table 1 depicts the surface characteristics of nanocel and SPEDA@nanocel hybrid material.

The results of Table 1 show that the grafting of TMSPEDA onto nanocellulose increased the HI value, making the hybrid material less hydrophilic than cellulose^55^. Although this phenomenon was observed, both have a high affinity to water compared to other materials^40,41,54,55^. This high affinity helps the hybrid material have contact with the polar RY-2 reactive dye (see Fig. S1)^29–31^.

The pH_pzc_ of the hybrid material (see Fig. S2 and Table 1) was shifted from 5.932 to 7.498, meaning that the grafting of SPEDA groups on the cellulose left the material with more alkaline behavior^29–31^. The hybrid material presents an expected isoelectric point of 9.12–9.40 (see Fig S3), which agrees with the pH_pzc._ Therefore, at pH < 9.3, the SPEDA@nanocel hybrid surface will present a positive charge, and below 9.3, it will be negatively charged^29–31^.

The BET surface areas of both materials are extremely low values (< 1 m^2^g^−1^) for both cellulosic materials. This result is critical to establish that the adsorption should take place based on the SPEDA groups inserted on the nanocel, and the pore-filling mechanism is totally disregarded. The pore-filling mechanism is expected to happen with materials presenting high surface area and total pore volume^54^.

### Optimization of initial pH

The effect of the initial RY-2 pH solution on SPEDA@nanocel adsorption capacity for the removal of RY-2 dye is depicted in Fig S4. As already expected, removing reactive dyes using protonated amino adsorbents is facilitated in low pH values^61–64^. From pH 2 to 7, the removal percentage decreases from 58.71 to 32.28%, and from pH 7 to pH 10, the removal percentage decreases up to 2.58%. The amino groups are protonated at low pH values, and the reactive dyes are negatively charged even at low pH values and are attracted to the positively charged adsorbent^27,31,61–64^. The results of the effect of initial pH are coherent with the pH_pzc_ (Fig S2). In order to continue this research, the initial pH of the RY-2 dye solution was fixed at 2.0.

### Kinetic study

**Fig. 7:**
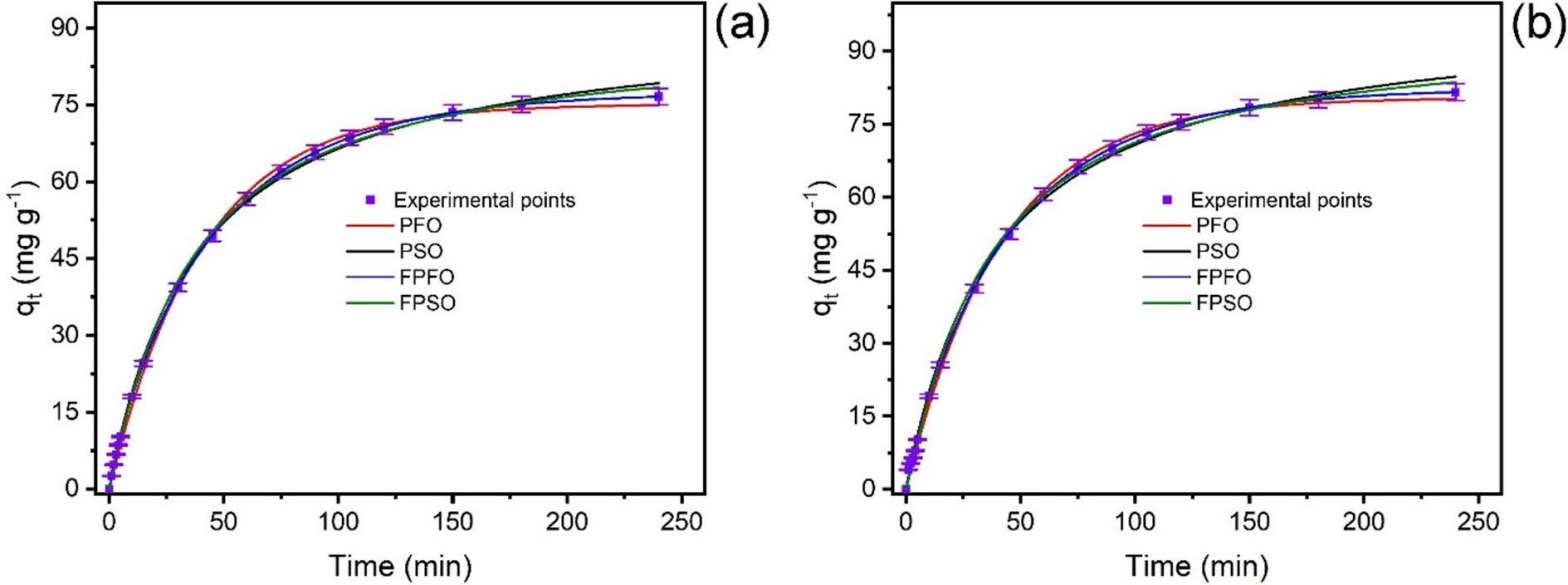
Adsorption kinetics of RY-2 onto SPEDA@nanocel material. (**a**) C_0_ = 250 mg L^−1^ RY-2; (**b**) C_o_ = 500 mg L^−1^ RY-2. Initial pH = 2.0, adsorbent mass = 30.0 mg, volume of dye = 20.00 mL, temperature = 25 °C.

The adsorption kinetic studies for the uptake of RY-2 onto SPEDA@nanocel were carried out using four kinetic models (Table 2, Fig. 7). The kinetic models were evaluated utilizing R^2^_adj_, SD, and BIC values^57,58^.

**Table 2:** Kinetic parameters for adsorption of RY-2 onto SPEDA@nanocel adsorbent. Conditions: SPEDA@nanocel mass of 30.0 mg, dye solution of 20.00 mL, pH = 2.0, 25 °C.

C_o_ (mg L^−1^)		
Pseudo-first order	250.0	500.0
q_e_ (mg g^−1^)	75.17	80.44
k_1_ (min^−1^)	0.02439	0.02391
t_1/2_ (min)	28.30	28.85
t_0.95_ (min)	120.66	122.80
R^2^ adjusted	0.9983	0.9987
SD (mg g^−1^)	1.214	1.146
BIC	13.53	11.46
Pseudo-second order		
q_e_ (mg g^−1^)	91.92	98.74
k_2_ (g mg^−1^ min^−1^)	2.836.10^−4^	2.561.10^−4^
t_1/2_ (min)	29.06	29.74
t_0.95_ (min)	173.7	174.9
R^2^ adjusted	0.9988	0.9981
SD (mg g^−1^)	1.033	1.384
BIC	7.708	18.25
Fractal-PFO order		
q_e_ (mg g^−1^)	77.44	82.37
k_1,0_ (min^−1^)	0.02266	0.02257
n	0.8930	0.9122
t_1/2_ (min)	28.77	29.21
t_0.95_ (min)	140.4	138.7
R^2^ adjusted	0.9999	0.9998
SD (mg g^−1^)	0.02254	0.4891
BIC	− 128.2	− 17.47
Fractal-PSO order		
q_e_ (mg g^−1^)	88.41	93.73
k_2,0_ (g mg^−1^ min^−n^)	2.493.10^−4^	2.135.10^−4^
n	1.073	1.100
t_1/2_ (min)	28.36	28.80
t_0.95_ (min)	167.8	167.1
R^2^ adjusted	0.9991	0.9987
SD (mg g^−1^)	0.8864	1.155
BIC	3.938	13.48

It was observed that the R_adj_^2^ values were close to 1.00, and the lowest SD values were obtained using the Fractal-like Pseudo-First-Order^57,60^. Notwithstanding, the ΔBIC values using two distinct models can be used to verify the best-fitted model.

When ΔBIC < 2, there is no remarkable difference between the two models^58^; for 2 < ΔBIC < 6, the model with a low BIC value tends to be the best-fitted model^58^; for 6 < ΔBIC < 10, the model with a low BIC value has a strong possibility of being the best-fitted model^58^; and ΔBIC ≥ 10 the model with the low value of BIC is certainly the best-fitted model^58^. The difference in BIC values between the different kinetic models and fractal-PFO ranged from 132.2 to 141.8 (250.0 mg L^−1^ RY-2) and 28.93–35.72 (500.0 mg L^−1^ RY-2). Therefore, there is no doubt from the statistical viewpoint that fractal-like pseudo-first-order is the best kinetic model to describe the uptake of RY-2 dye using SPEDA@nanocel adsorbent^57,58^.

Observing the kinetic constant rates of different models in Table 2 presents different units, making it challenging to compare these models^40,54^. Conversely, the time to attain 50% saturation (t_0.5_) and 95% saturation (t_0.95_) of the adsorbent becomes a powerful tool to compare different kinetic models^40,54^. Therefore, based on the fractal-PFO, the t_0.5_ and t_0.95_ ranged from 28.71–29.21 min and 138.7–140.4 min, respectively. The adsorption kinetics of RY-2 onto SPEDA@nanocel is not a fast kinetic compared to adsorbent materials with high surface area and total pore volumes^54,65–67^. For continuing this work, the contact time between the adsorbent and adsorbate was fixed at 180 min to guarantee complete contact between adsorbent and adsorbate to attain equilibrium^37,38,40,54,57^.

The intraparticle diffusion model^68^ was also applied to the system of RY-2 dye and SPEDA@nanocel adsorbent (Fig S5)—the plot q_t_ *vs.* $\sqrt{t}$ presented three linear sections, which indicate that the intraparticle diffusion is not the only mechanism of uptake of RY-2 onto SPEDA@nanocel hybrid material^32,68^. The first linear step is assigned to the diffusion of the dye into the film, which involves the solid particles^32,68^. The second linear step is the intraparticle diffusion^32,68^. Finally, the third linear section corresponds to the diffusion of the adsorbate through the smaller pores of the adsorbent and the binding with the active sites of the adsorbent^32,68^. The intraparticle diffusion constants of the second linear section (k_2, id_) were 3.868 (250 mg L^−1^ RY-2) and 4.023 mg g^−1^ min^−0.5^ (500 mg L^−1^ RY-2).

### Equilibrium, thermodynamics, and mechanism of adsorption

**Fig. 8:**
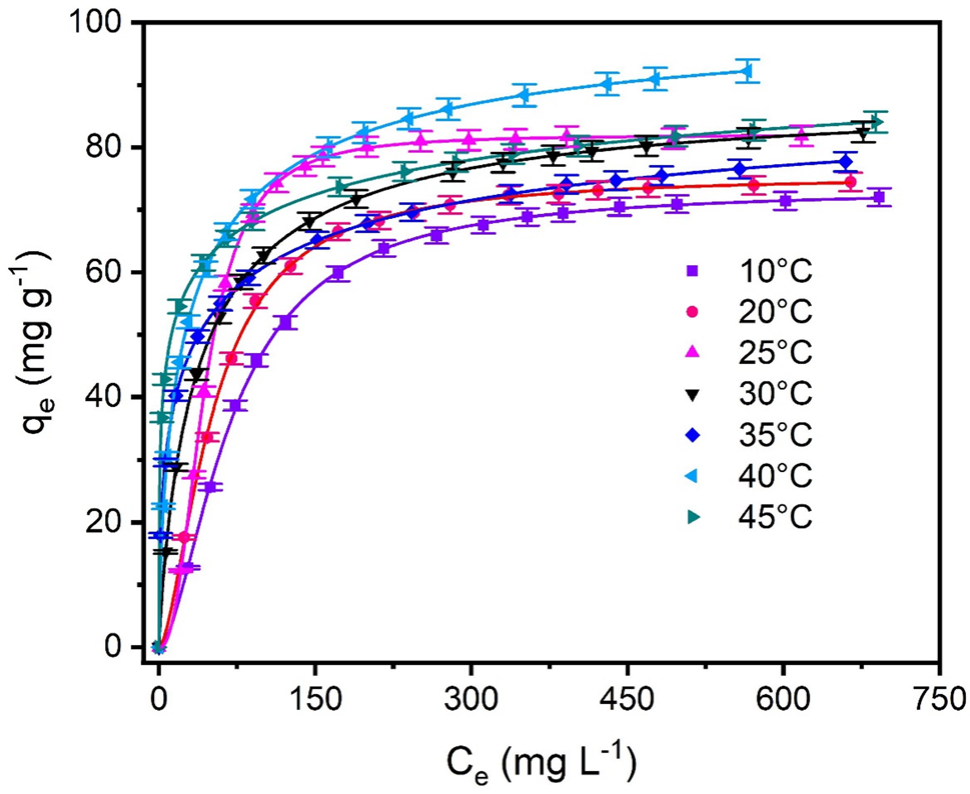
Isotherms of adsorption of RY-2 onto SPEDA@nanocel adsorbent. The curves were fitted to Liu’s model. Conditions: Contact time = 180 min; pH = 2.0; adsorbent mass = 30.0 mg; dye solution = 20.00 mL.

The equilibrium studies of RY-2 dye onto SPEDA@nanocel adsorbent were carried out from 10 to 45 °C using three equilibrium models (Table 3, Fig. 8).

**Table 3:** Langmuir, Freundlich, and Liu isotherm parameters for RY-2 uptake using SPEDA@nanocel.

Langmuir	10 °C	20 °C	25 °C	30 °C	35 °C	40 °C	45 °C
*Q*_max_ (mg g^−1^)	85.09	84.33	96.06	85.78	74.76	92.18	78.51
*K*_L_ (L mg^−1^)	0.01134	0.01712	0.01956	0.02856	0.07188	0.04940	0.2038
*R*^2^_adj_	0.9800	0.9818	0.9330	0.9987	0.9589	0.9876	0.9314
SD (mg g^−1^)	3.230	3.045	7.086	0.9213	4.742	3.115	5.970
BIC	43.70	41.81	68.84	3.560	55.98	42.55	63.65

Freundlich	10 °C	20 °C	25 °C	30 °C	35 °C	40 °C	45 °C
*K*F(mg.g^−1^ (mg L-1)-1/nF)	10.01	15.17	17.44	18.43	23.64	24.23	36.33
*n*_F_	3.122	3.817	3.768	4.109	5.228	4.505	7.582
*R*^2^_adj_	0.9022	0.8971	0.8022	0.9465	0.9801	0.9651	0.9920
SD (mg g^−1^)	7.142	7.236	12.17	5.907	3.298	5.233	2.042
BIC	69.09	69.51	86.15	63.02	44.37	59.14	29.03

Liu	10 °C	20 °C	25 °C	30 °C	35 °C	40 °C	45 °C
*Q*_max_ (mg g^−1^)	73.43	75.53	82.02	88.90	96.19	104.1	112.6
*K*_g_ (L mg^−1^)	0.01424	0.01929	0.02289	0.026616	0.03091	0.03590	0.04170
*n*_L_	1.674	1.625	2.385	0.8836	0.4783	0.6815	0.3217
*R*^2^_adj_	0.9997	0.9996	0.9998	0.9999	0.9999	0.9999	0.9999
SD (mg g^−1^)	0.3638	0.4564	0.3707	0.004542	0.08759	0.003204	0.003985
BIC	− 24.58	− 17.34	− 23.99	− 164.9	− 70.15	− 176.0	− 169.0

Conditions: initial pH = 2.0, contact time = 180 min, adsorbent mass = 30.0 mg, RY-2 volume = 20.00 mL.

The Langmuir, Freundlich, and Liu equilibrium models fitted the experimental data (10^–^45 °C)^57^. Statistically, the Liu isotherm model was the best-fitted equilibrium model since the R^2^_adj_ values were closer to 1, and also, this model presented the lowest SD values than the Langmuir and Freundlich model^39,40^. Although good values of R^2^_adj_ and lowest values of SD are not a guarantee of choosing the best-fitted model, the BIC is the best Statistical evaluation to choose the best model that describes the phenomena^57,58^. As previously described, when ΔBIC between two models is ≥ 10, unquestionably, the model with the lowest SD values is the best choice to describe the phenomena^59,60^. The difference between BIC values between Langmuir and Liu models ranged from 59.15 to 232.4, and Freundlich and Liu ranged from 86.85 to 235.2. These ΔBIC are much higher than 10, which means that there is no doubt that the Liu isotherm model is the best model (10–45 °C) to describe the adsorption equilibrium of RY-2 onto SPEDA@nanocel hybrid adsorbent^40,54,57,58^.

The Q_max_ based on the Liu isotherm model ranged from 73.43 to 112.6 mg g^−1^ of RY-2 adsorbed on SPEDA@nanocel material, with the maximum value attained at 45 °C. The maximum sorption capacities of several adsorbents were compared to the uptake of Reactive Yellow 2 (or Cibacrom Brilliant Yellow 3G-P, which is another commercial tradename)^64,69–84^.

According to the results depicted in Table 4, the MCC adsorbent presents a higher sorption capacity for the adsorption of RY-2, which is ranked as 12 out of 30 adsorbents^64,69–84^, and 17 out of 30 adsorbents presented higher sorption capacity to the uptake of RY-2 when compared to MCC material. This result shows that the sorption of the MCC is moderated when compared with values already reported in the literature. Also, it is remarkable to state that in 5 out of 17 adsorbents that presented sorption capacity higher than MCC, the results of Q_max_ were obtained using linear fitting, and these values could be overestimated^57,85,86^. Also, it could be stated that one of the reasons for the MCC medium sorption capacity is the low surface area of this adsorbent (0.4 m^2^ g^−1^). The primary mechanism of RY-2 should occur only at the external surface of the material, and the pore-filling mechanism should be ruled out^54,85,86^.

**Table 4:** Values of Q_max_ for the uptake of RY-2 onto different adsorbent materials.

Adsorbent	Q_max_ (mg g^−1^)	Fitting	^REF^
Magnetized chitosan beads (MC)	131.58	Linear	^69^
MC coated with tetraethyl orthosilicate TEOS (TMC)	178.57	Linear	^69^
MC modified with TEOS and ethylenediamine (ETMC)	243.90	Linear	^69^
Chitosan	4.849	Linear	^70^
Polyvinylpyrrolidone-chitosan [PVP-CS]	6.313	Linear	^70^
Poly(vinyl alcohol)-chitosan [PVA-CS]	6.988	Linear	^70^
2-Hydroxypropyl-β-cyclodextrin-chitosan [HPβCD-CS]	8.818	Linear	^70^
Poly (vinyl alcohol)-polyvinylpyrrolidone-chitosan [PVA-PVP-CS]	6.761	Linear	^70^
Polyamide-chitosan-citric acid cross-linked	37.04	Linear	^71^
Sn-doped TiO_2_ on activated carbon	104	Linear	^72^
Polypyrole (PPy), chitosan (CS), and Sn-doped Ti	103	Linear	^72^
High-surface-area mesoporous MgO-templated nanocarbon	293.2	Nonlinear	^73^
High-surface-area mesoporous MgO-templated nanocarbon	269.0	Nonlinear	^73^
Commercial activated carbon	495.5	Nonlinear	^73^
Graphene-Oxide–Chitosan Aerogel composites	748.8	Nonlinear	^74^
TiO_2_ degusa	3.86	Linear	^75^
TiO_2_ anatase/Diphenylcarbizide	4.64	Linear	^75^
Fe_2_O_3_	8.61	Linear	^75^
Polyethylenimine-3-Aminopropyltriethoxysilane-MWCNT	742.4	Linear	^64^
Activated sludge	333.3	Linear	^76^
Waste biomass from the lysine fermentation process	178.5	Nonlinear	^77^
Commercial activated carbon	209.5	Nonlinear	^78^
Tetra-*n*-butylammonium bromide-modified sugar beet pulp	62.85	Linear	^79^
Polyethylenimine/Polyvinyl Chloride Cross-Linked Fiber	820.6	Nonlinear	^80^
Immobilized *Gibberella fujikuroi* on maize tassel biomatrix	379.7	Nonlinear	^81^
Polyurethane-immobilized on *Corynebacterium glutamicum*	116.5	Nonlinear	^82^
Polysulfone/bacterial biomass	153.2	Nonlinear	^83^
Polyethylenimine-coated polysulfone/bacterial biomass	586.8	Nonlinear	^83^
Polyethylenimine-crosslinked calcium silicate hydrate	235.0	Nonlinear	^84^
Magnetic carbon composite	112.6	Nonlinear	This work

The thermodynamic adsorption parameters were calculated using the nonlinear van't Hoff equation (Fig S6, Table 5)^57^ using the values of Liu's isotherm equilibrium constant (K_g_) that were converted to the dimensionless thermodynamic equilibrium constant (${K}_{e}^{0}$), according to previously reported^59^. For details, see Supplementary Material.

**Table 5:** Thermodynamic adsorption parameters.

T (K)	283	293	298	303	308	313	318
${K}_{e}^{0}$(L mol^−1^)	1.243.10^4^	1.684.10^4^	1.998.10^4^	2.323.10^4^	2.698.10^4^	3.134.10^4^	3.640.10^4^
ΔG° (kJ mol^−1^)	− 22.18	− 23.71	− 24.53	− 25.33	− 26.13	− 26.94	− 27.77
ΔS° (J K^−1^ mol^−1^)	160.97						
ΔH° (kJ mol^−1^)	23.44						
R^2^	0.9995						
R^2^_adj_	0.9994						

The ${K}_{e}^{0}$ was calculated according to the literature ^60^ based on the Liu isotherm model ranging from 283 to 318 K.

The ΔG° values were always < 0, suggesting that the adsorption process was favorable^57,59^. The ΔH° was positive, indicating that the process of adsorption was endothermic (Fig S6), and the ΔH° magnitude (23.44 kJ mol^−1^) is consistent with a physical adsorption process^57,87^. The positive value of ΔS° is compatible with the RY-2 molecule initially hydrated, which lost its hydrated water to the system before the dye was uptaken by the SPEDA@nanocel hybrid material^88^.

**Fig. 9:**
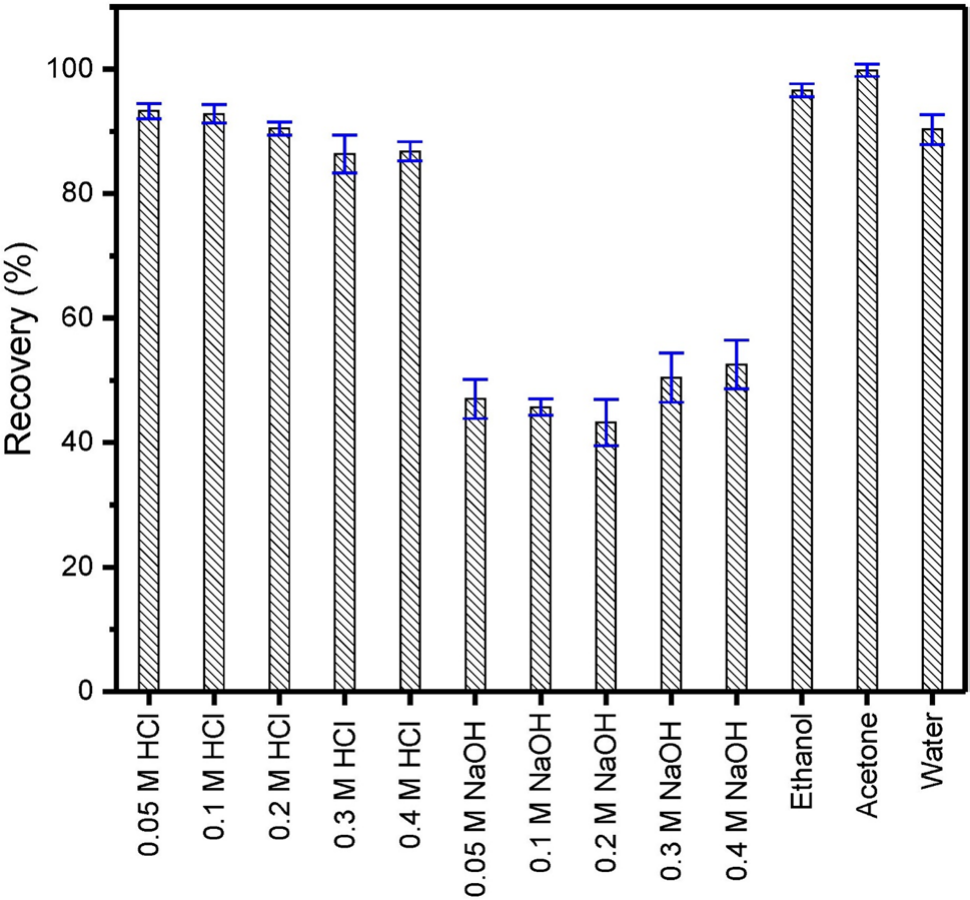
Five consecutive cycles of adsorption and desorption of RY-2 onto SPEDA@nanocel.

Five consecutive cycles of adsorption/desorption of RY-2 onto SPEDA@nanocel were carried out with the following eluents: 0.05–0.4 M HCl, 0.05–0.4 M NaOH, ethanol, acetone, and water (Fig. 9). The best eluent was acetone (recovery > 99%), followed by ethanol (recovery > 96%), and then by water (recovery > 90%). For the aqueous solutions, HCl recoveries ranged from 86 to 93%, and the worst eluent was NaOH solutions (43–52%). It is important to note that after five cycles of adsorption and desorption, a decrease in the sorption capacity of SPEDA@nanocel was not noticed. Also, the repeatability of the results was excellent, with standard deviations < 4%.

**Fig. 10:**
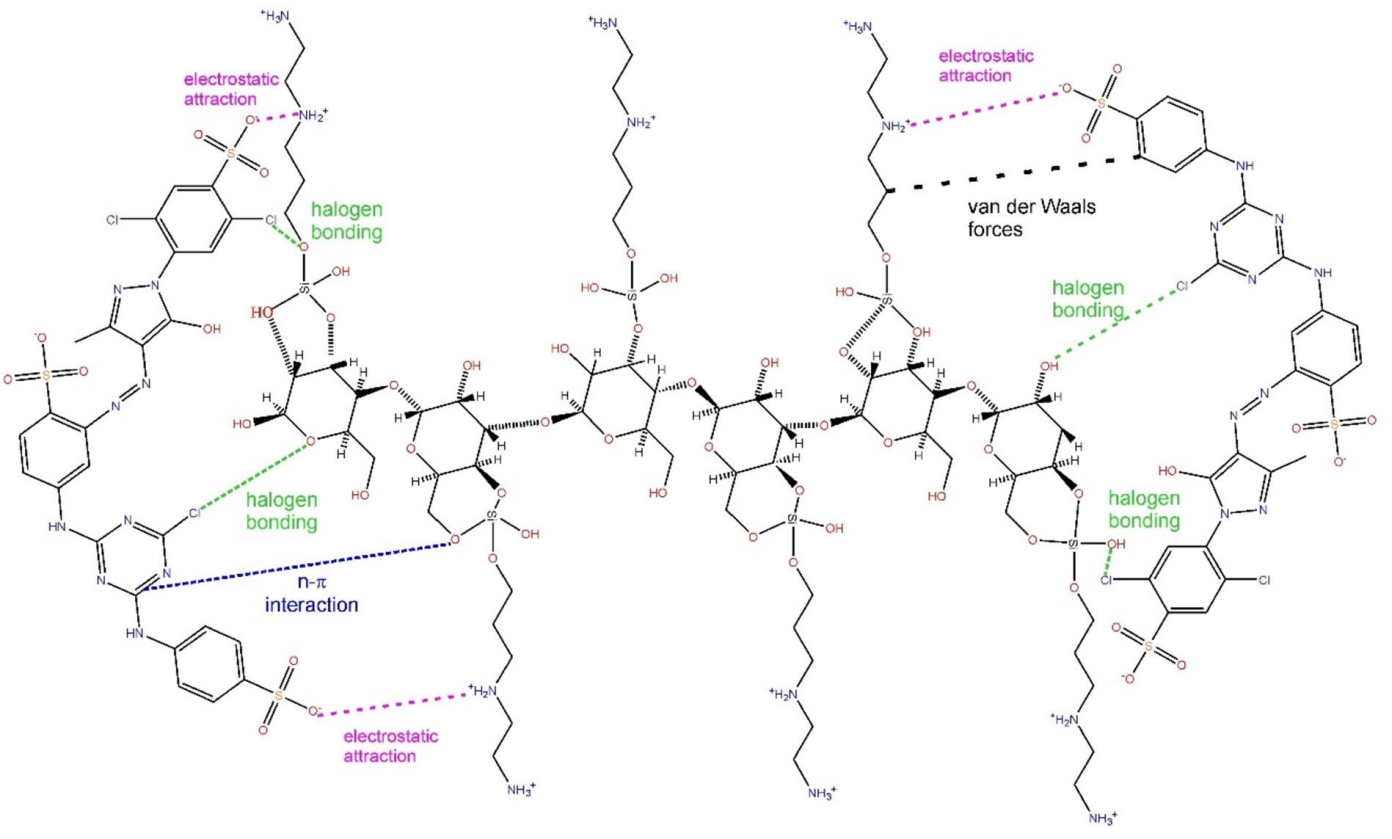
Interaction mechanism of RY-2 onto SPEDA@nanocel material.

Based on the characterization of the material (surface area, functional groups, pH_pzc_) and adsorption studies (effect of initial pH of RY-2 solution, kinetics, equilibrium, thermodynamics, and recovery), a mechanism of adsorption of RY-2 onto SPEDA@nanocel can be established. The hybrid material presents a BET surface area lower than 1 m^2^ g^−1^; this suggests that the pore-filling mechanism should be disregarded. Taking into account the pH_pzc_ of hybrid material and the effect of initial pH, that suggests that pH 2.0 would be the best for the uptake of RY-2, and also considering that the kinetics is not so fast (because it should occur only at the external surface of the adsorbent, owing to low surface area and pore volume) and the magnitude of ΔH° (compatible with physical adsorption), the mechanism of uptake of RY-2 should be mainly electrostatic attraction^27–31,88^ and some halogen bonding^89^ (Fig. 10). Other n-π interactions also could take place^41^ owing to the interactions of the organics parts of the dye with the adsorbent^41^. Figure 10 depicts the possible interaction mechanism of RY-2 dye with SPEDA@nanocel hybrid material.

## Conclusions

A successful hybrid material was prepared by grafting TMSPEDA onto nanocellulose material. SEM, SEM–EDS, FTIR, XRD, TGA, HI, and pH_pzc_ characterized the SPEDA@nanocel hybrid_._ The amount of inserted SPEDA moieties on the hybrid was 0.811 mmol g^−1^. The aminated hybrid material in low pH values presents all the amino groups protonated (pH_pzc_ 9.309). Considering that the pK_a_ values of RY-2 dyes are below zero because they are derivatives of sulfonic acid, the dye presents at least 3 formal charges (from the sulfonate groups) even at low pH values. Also, considering that the surface area of SPEDA@nanocel is irrisory, the primary adsorption mechanism should be the electrostatic attraction of the negatively charged dye (RY-2) with the positively charged adsorbent at pH 2.0 taking place at the external surface of the hybrid adsorbent. The adsorption experiments also confirmed this interaction mechanism. The adsorption kinetics do not present fast kinetics since t_0.95_ varies from 138.7 to 140.4 min, which is compatible with materials that do not have a high surface area.

The maximum sorption capacity of MCC at 318 K (45 °C) was 112.6 mg g^−1^_;_ it is a good but not outstanding value of sorption capacity when compared to other adsorbents.

Based on the Liu isotherm model (283–318 K), the thermodynamics experiments presented ΔH° of + 23.44 kJ mol^−1^ (whose magnitude is compatible with physical adsorption). Based on all the experimental results, a mechanism of adsorption was proposed that comprises electrostatic attraction, halogen bonding, and n-π (interaction) (all physical interactions). Experiments of recyclability of the adsorbent were performed, and the data showed that the adsorbent could be regenerated using HCl solutions, ethanol, water, and acetone. The SPEDA@nanocel hybrid material could be reutilized for 5 adsorption cycles without a decrease in the sorption capacity and presenting elevated repeatability between the cycles (standard deviations < 4%).

From a future perspective, other adsorbents with different numbers of amino groups in the organosilane could result in materials with higher sorption capacities. Also, the insertion of amine groups on other carbon supports by organo-silylation could lead to a generation of adsorbents with higher sorption capacity and fast uptake kinetics.

### Supplementary Information

Supplementary Information.

## Data Availability

The data that support the findings of this study are available from the corresponding author upon reasonable request.
