# Supplementary material for: Hybrid nanocellulose material as an adsorbent to remove reactive yellow 2 dye
Source: Sci Rep. 2024 Aug 29;14:20074. doi: 10.1038/s41598-024-70906-5 (PMC11362320; doi:10.1038/s41598-024-70906-5)
Supplement: Supplementary file 1 — Supplementary Information. [file 41598_2024_70906_MOESM1_ESM.pdf]

Supplementary Material

Hybrid nanocellulose material as an adsorbent to remove reactive yellow 2 dye.

2.4 Adsorption experiments

An aliquot of 20.00 mL of Reactive Yellow 2 (RY-2) dye solution with the initial concentration variating from 30.0 to 900.0 mg L^{-1} was added to 50.0 mL flat-Falcon tubes with 30.0 mg SPEDA@nanocell hybrid material at pH 2.0. The Falcon tubes were capped and disposed of horizontally inside a thermostatic reciprocating agitator (Oxy 350, São Leopoldo, Brazil). The slurries were shaken at different time intervals between 1 and 240 min at 10° to 45°C with a shaking speed of 120 strikes per minute [39,55,56]. Subsequently, the solid phase was separated from the liquid phase by centrifugation. When necessary, aliquots of 1-10 ml of the liquid phase were diluted to 1.0-25.0 mL in calibrated volumetric flasks using the blank solution. The dyes unadsorbed after the adsorption process were measured using the T90+ PG Instruments spectrophotometer at a maximum absorption wavelength of 404 nm (RY-2).

The sorption capacity (Eq 1) and the percentage of adsorbate removed (Eq 2) are given below:

$$q = \frac{(C_0 - C_f)}{m}.V \quad (1)$$

$$\% \, Removal = 100.\frac{(C_0 - C_f)}{C_0} \quad (2)$$

q is the sorption capacity of adsorbate adsorbed by the adsorbent (mg g^{-1}). C_o is the initial adsorbate solution concentration in contact with the solid adsorbent (mg L^{-1}). C_f is the

final adsorbate concentration after adsorption (mg L^{-1}). *m* is the mass of adsorbent (g). *V* is the aliquot of the adsorbate solution (L) introduced in the flask.

The study of the influence of the initial pH of adsorbate was performed at 25°C, using an initial concentration of 300 mg L^{-1} of RY-2, a time of contact between the adsorbent and adsorbates of 2 h, an adsorbent dosage of 1.5 g L^{-1}, and pH 2.0-10.0.

The preliminary experiments were conducted to ensure the experimental data's reproducibility, reliability, and accuracy. The relative standard deviations of all measurements were below 3.5% [58,61]. Blanks were run in parallel and corrected when necessary [55].

The solutions of adsorbates were stored in glass bottles, cleaned, rinsed with deionized water, dried, and stored in a suitable cabinet [55].

Standard RY-2 solutions (1.0-100.0 mg L^{-1}) were calibrated in parallel with a blank. The linear analytical calibration curve was performed on the UV-Win software of the T90+ PG Instruments spectrophotometer. The detection limit of RY-2 was 0.28 mg L^{-1}, with a signal/noise ratio of 3 [55].

A 5.0 mg L^{-1} of standard RY-2 solutions was used as quality control after every ten measurements to ensure the accuracy of the analytes measurements [55].

The kinetic and equilibrium data's fitness was done using nonlinear methods, which were evaluated using the Simplex method first and secondly by the Levenberg–Marquardt algorithm using the fitting facilities of the Microcal Origin 2021 software [58]. The suitableness of the kinetic and equilibrium models was evaluated using the residual sum of squares (*RSS*), the determination coefficient (R^2), the adjusted determination coefficient (R^2_{adj}), the standard deviation of residues (*SD*), and the Bayesian Information Criterion (*BIC*) [58,61]. Equations 3 to 7 are the mathematical expressions for respective *RSS*, R^2, R^2_{adj}, *SD, and BIC*.

$$RSS = \sum_{i}^{n} \left(q_{i,exp} - q_{i,model}\right)^2 \tag{3}$$

$$R^2 = \left(\frac{\sum_i^n \left(q_{i,exp} - \overline{q}_{i,exp}\right)^2 - \sum_i^n \left(q_{i,exp} - q_{i,model}\right)^2}{\sum_i^n \left(q_{i,exp} - \overline{q}_{i,exp}\right)^2} \right) \tag{4}$$

$$R^2_{adj} = 1 - (1 - R^2).\left(\frac{n-1}{n-p-1}\right) \tag{5}$$

$$SD = \sqrt{\left(\frac{1}{n-p}\right).\sum_{i}^{n} \left(q_{i,exp} - q_{i,model}\right)^2} \tag{6}$$

$$BIC = nLn\left(\frac{RSS}{n}\right) + pLn(n) \tag{7}$$

In the above equations, the $q_{i,\ model}$ is the individual theoretical q value predicted by the model; $q_{i,\ exp}$ is individual experimental q value; $\overline{q}_{i,exp}$ is the average of all experimental q values measured; n is the number of experiments; p is the number of parameters in the fitting model.

The values of R^2_{adj}, SD, and BIC will be presented to compare different models of kinetics and equilibrium presented in this work. The best-fitted model would present R^2_{adj} closer to 1.000, lower values of SD, and BIC values. However, the kinetic and equilibrium models could not merely be chosen based on the values of R^2 [58] when these models present a different number of parameters. Therefore, it is necessary to check if the improvements in the R^2 values are due to the increase in the parameters [58] or if, physically, the model with more parameters better explains the process taking place [58].

However, the difference in BIC values between models could be conclusive if the difference in BIC values ≤ 2.0) shows no significant difference between the two models [58,61]. When the difference in BIC values is between 2 and 6, there is a positive perspective that the model with lower BIC is the most suitable [58,61]. For variations of BIC values from 6-10, there is

a strong possibility that the model with a lower BIC value would be the best model to be fitted [58,61]. However, if the difference in BIC values ≥ 10.0, it can be predicted with accuracy that the model with a lower BIC value is better fitted [58,61].

Kinetic, equilibrium, and thermodynamic studies

The kinetic adsorption data were evaluated by using four nonlinear models: pseudo-first-order (PFO) [58], pseudo-second-order PSO [58], fractal-like pseudo-first-order (FL-PFO) [59], and fractal-like pseudo-second-order (FL-PSO) [59]. The mathematical equations of these respective models are shown in Equations 8-11.

$$q_t = q_e.[1 - exp\,(-k_1.t)] \tag{8}$$

$$q_t = \frac{k_2.q_e^2.t}{1+q_e.k_2.t} \tag{9}$$

$$q_t = q_e.[1 - exp\,(-k_{1,0}.t)^n] \tag{10}$$

$$q_t = \frac{k_2.q_e^2.t^n}{1+q_e.k_{2,0}.t^n} \tag{11}$$

Where t is the contact time (min); q_t, and q_e are the amount of adsorbate adsorbed at time t and the equilibrium, respectively (mg g^{-1}); k_1 is the pseudo-first-order rate constant (min^{-1}); k_2 is the pseudo-second-order rate constant (g mg^{-1} min^{-1}); $k_{1,0}$ is the fractal-like pseudo-first-order constant rate (min^{-1}), $k_{2,0}$ is the fractal-like pseudo-second-order rate constant (g mg^{-1} min^{-n}), and n is the fractional-like exponent (n >0).

Langmuir, Freundlich, and Liu's models were employed to analyze equilibrium data.

Equations 12, 13, and 14 show the corresponding Langmuir [58], Freundlich [58], and Liu models [58].

$$q_e = \frac{Q_{max}.K_L.C_e}{1 + K_L.C_e} \tag{12}$$

$$q_e = K_F.C_e^{1/nF} \tag{13}$$

$$q_e = \frac{Q_{max}.(K_g.C_e)^{nL}}{1+(K_g.C_e)^{nL}} \tag{14}$$

Where q_e is the adsorbate amount adsorbed at equilibrium (mg g^{-1}); C_e is the adsorbate concentration at equilibrium (mg L^{-1}); Q_{max} is the maximum sorption capacity of the adsorbent (mg g^{-1}); K_L is the Langmuir equilibrium constant (L mg^{-1}); K_F is the Freundlich equilibrium constant [mg.g^{-1}.(mg.L^{-1})$^{-1/nF}$]; K_g is the Liu equilibrium constant (L mg^{-1}); n_F and n_L are the exponents of Freundlich and Liu model, respectively, (n_F and n_L are dimensionless).

Thermodynamic studies for the adsorption of RY-2 dye onto *SPEDA@nanocell* were performed at a temperature ranging from 10ºC to 45°C (283 to 318K).

The Gibb's free energy change ($\Delta G°$, kJ mol^{-1}), enthalpy change ($\Delta H°$, kJ mol^{-1}), and entropy change ($\Delta S°$, J mol^{-1}K^{-1}) were evaluated with the aid of Equations 15-19, respectively [60].

$$\Delta G^{\Theta} = \Delta H^0 - T.\Delta S^0 \tag{15}$$

$$\Delta G^0 = -RT.LnK_e^0 \tag{16}$$

$$K_e^0 = \frac{(1000.K_g.Mw.[adsorbate]^0}{\gamma} \tag{17}$$

The combination of Equations 15 and 16 leads to equation 18

$$LnK_e^0 = \frac{\Delta S^0}{R} - \frac{\Delta H^0}{R}.\frac{1}{T} \tag{18}$$

The nonlinear form of equation 18 is [60]:

$$K_e^0 = exp\left[\frac{\Delta S^0}{R} - \left(\frac{\Delta H^0}{R}\right) \cdot \frac{1}{T}\right] \quad (19)$$

Where R is the universal gas constant (8.314 J K^{-1} mol^{-1}); T is the absolute temperature (Kelvin); K_e^0 is the thermodynamic equilibrium constant, which was calculated according to equation 16. K_e^0 is dimensionless.

K_e^0 is calculated by converting the values of K_g (Liu equilibrium constant) or K_L (Langmuir equilibrium constant), which is expressed in L mg^{-1} into L mol^{-1}. Firstly, the value K_g or K_L is multiplied by 1000 (mg g^{-1}), and then multiplied by the molecular weight of the adsorbate (g mol^{-1}) and by the standard concentration of the adsorbate (1 mol L^{-1}) and divided by the activity coefficient of the adsorbate (dimensionless) [58,60]. The solution is assumed to be sufficiently diluted to consider that the activity coefficient is unitary. Making these calculations, K_e^0 becomes dimensionless [58,60].

Equation 19 was used for calculating ΔH° and ΔS°, and Equation 16 was used for calculating ΔG°.

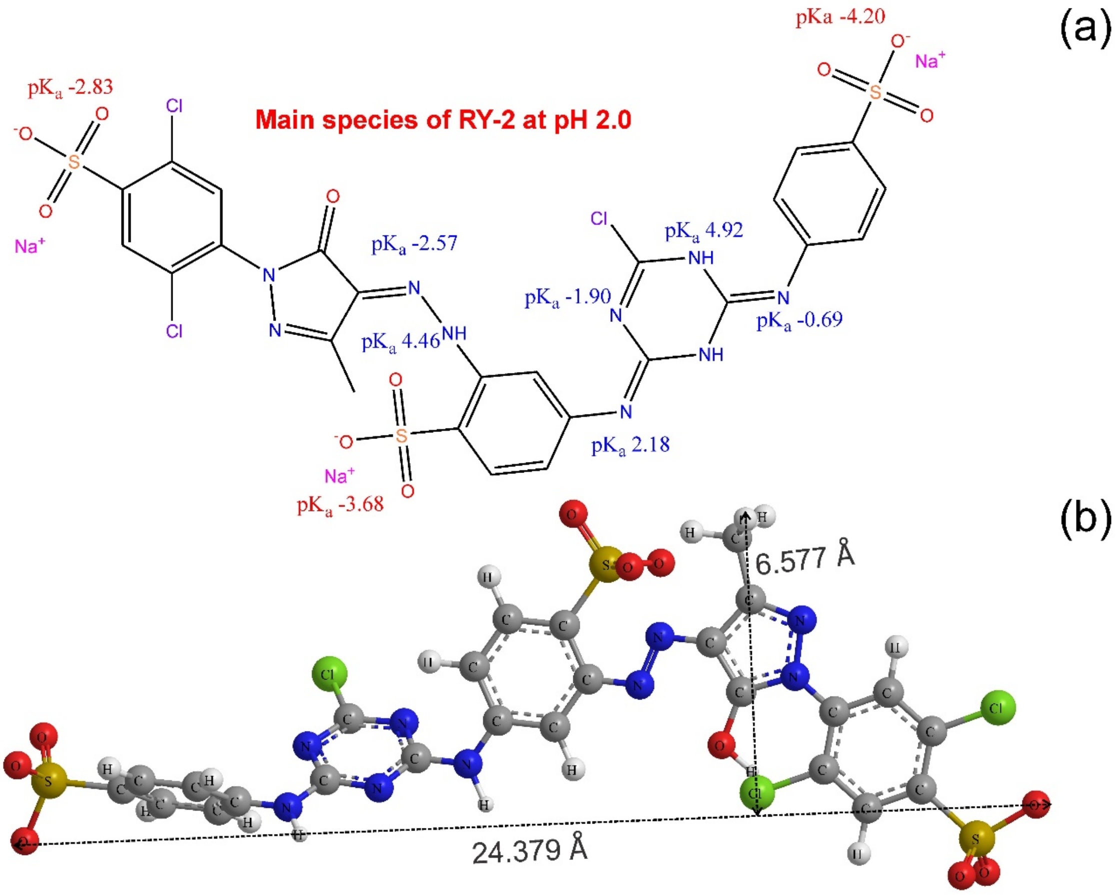

Fig S1. (a) Structural formula of Reactive Yellow 2 (RY-2; 872.947 g mol^{-1}; $C_{25}H_{15}N_9O_{10}S_3Cl_3Na_3$). pK_a values are given. (b) The optimized 3D structural formula of RY-2. The dimensions of the chemical molecule and physical-chemical properties were calculated using MarvinSketch version 24.1.2. Dipole Moment 281.79 Debye; van der Waals volume 566.32 Å^3; Polar surface area 322.24 Å^2; Van der Waals surface area (3D) 865.96 Å; HLB 36.80.

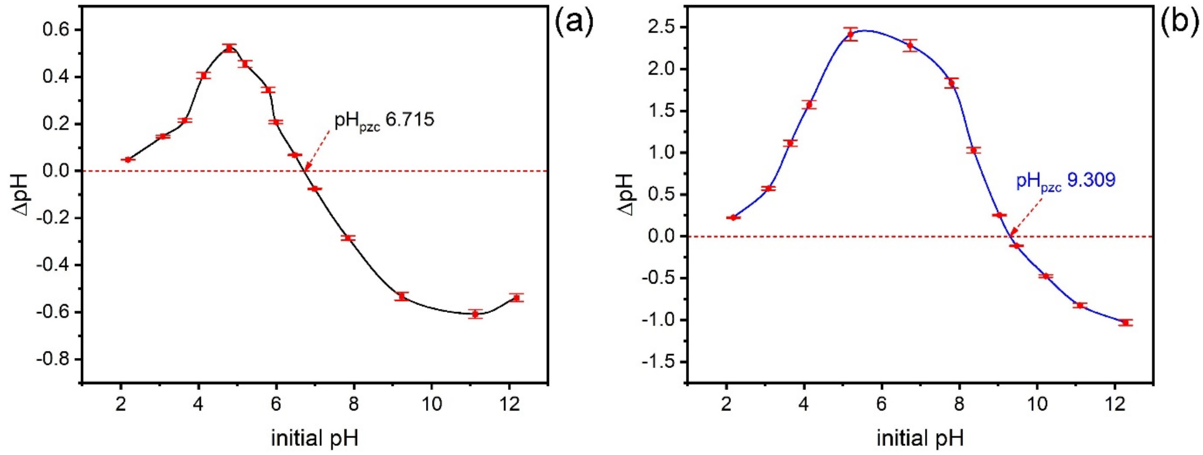

Fig S2. pHpzc of (a) nanocel, (b) SPEDA@nanocel.

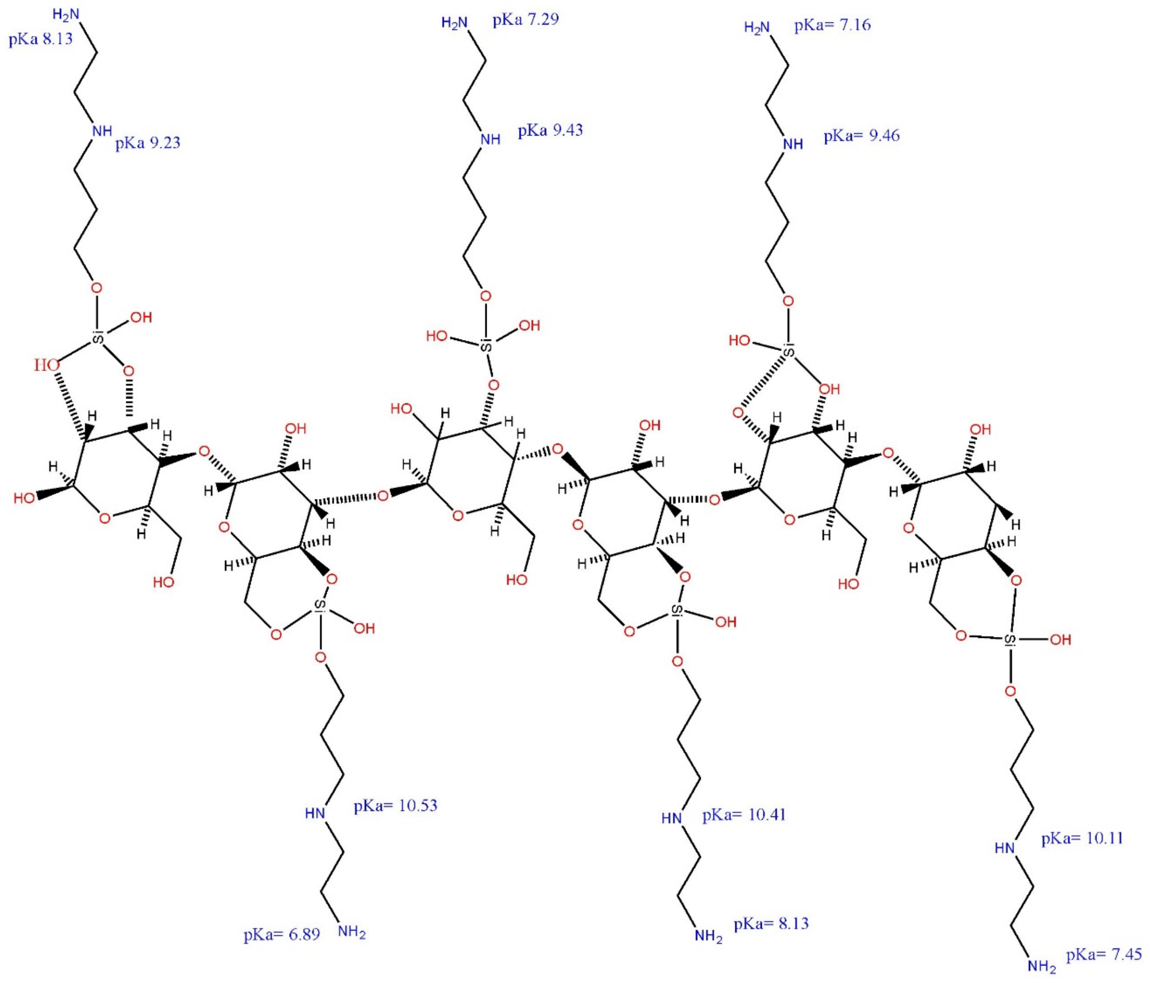

Fig S3. Estimated values of pKa of SPEDA@nanocel hybrid material and estimated isoeletric point of 9.12-9.40. Values were calculated using MarvinSketch version 24.1.2.

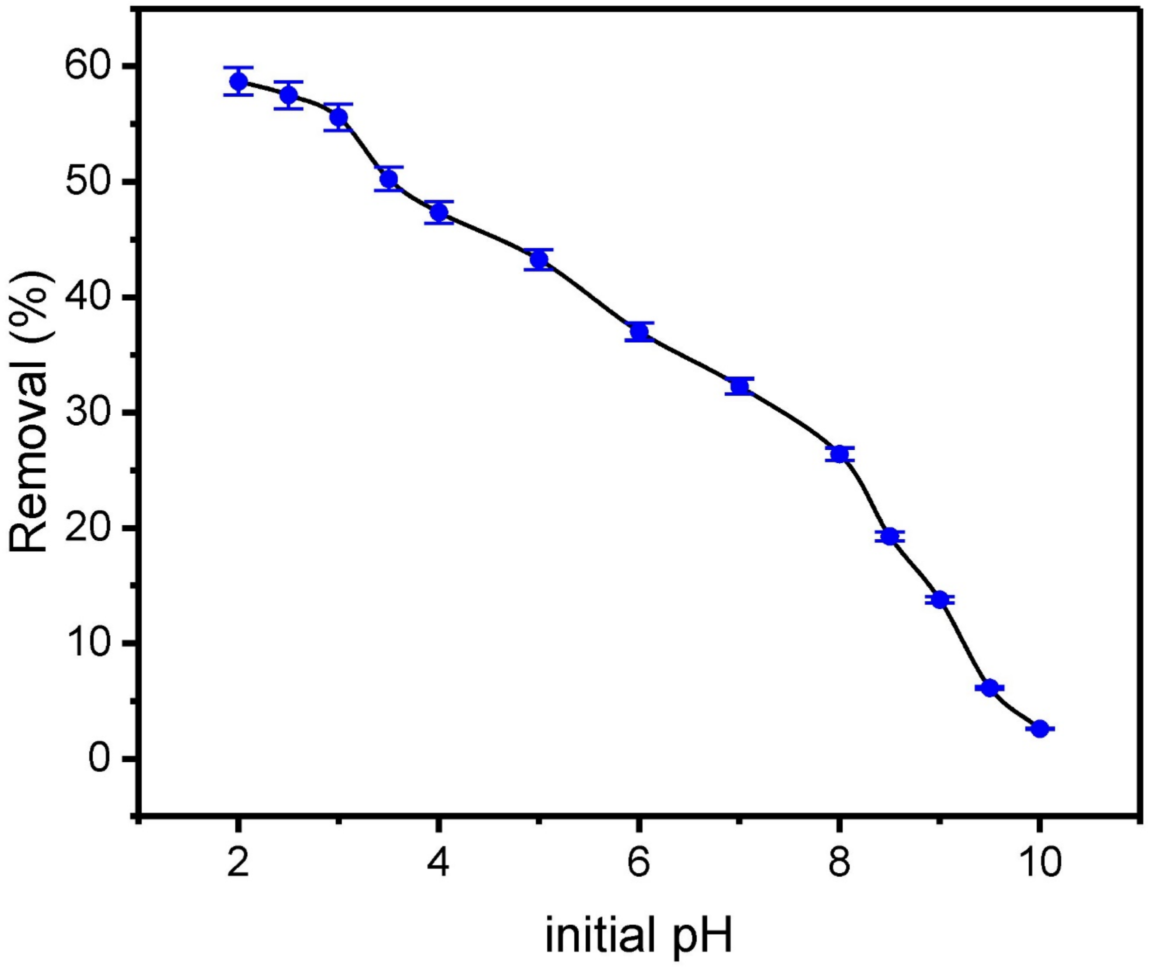

Fig S4. Effect of initial pH on the adsorption of RY-2 dye onto SPEDA@nanocel. Initial concentration 100 mg L^{-1}, 30.0 mg adsorbent, 20.00 mL dye solution, 25°C.

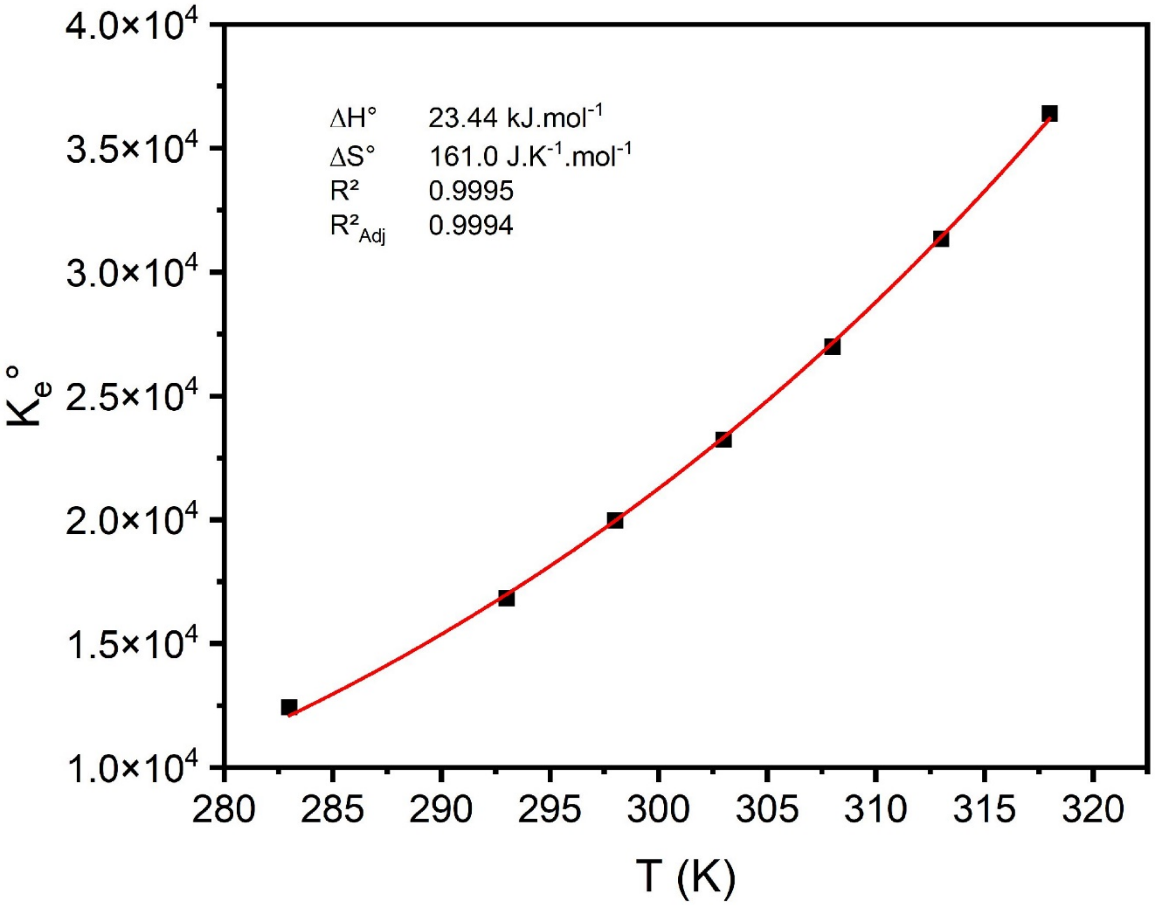

Fig S5. Nonlinear van´t Hoff curve for determination of thermodynamic adsorption parameters for the uptake of RY-2 onto SPEDA@nanocel adsorbent.

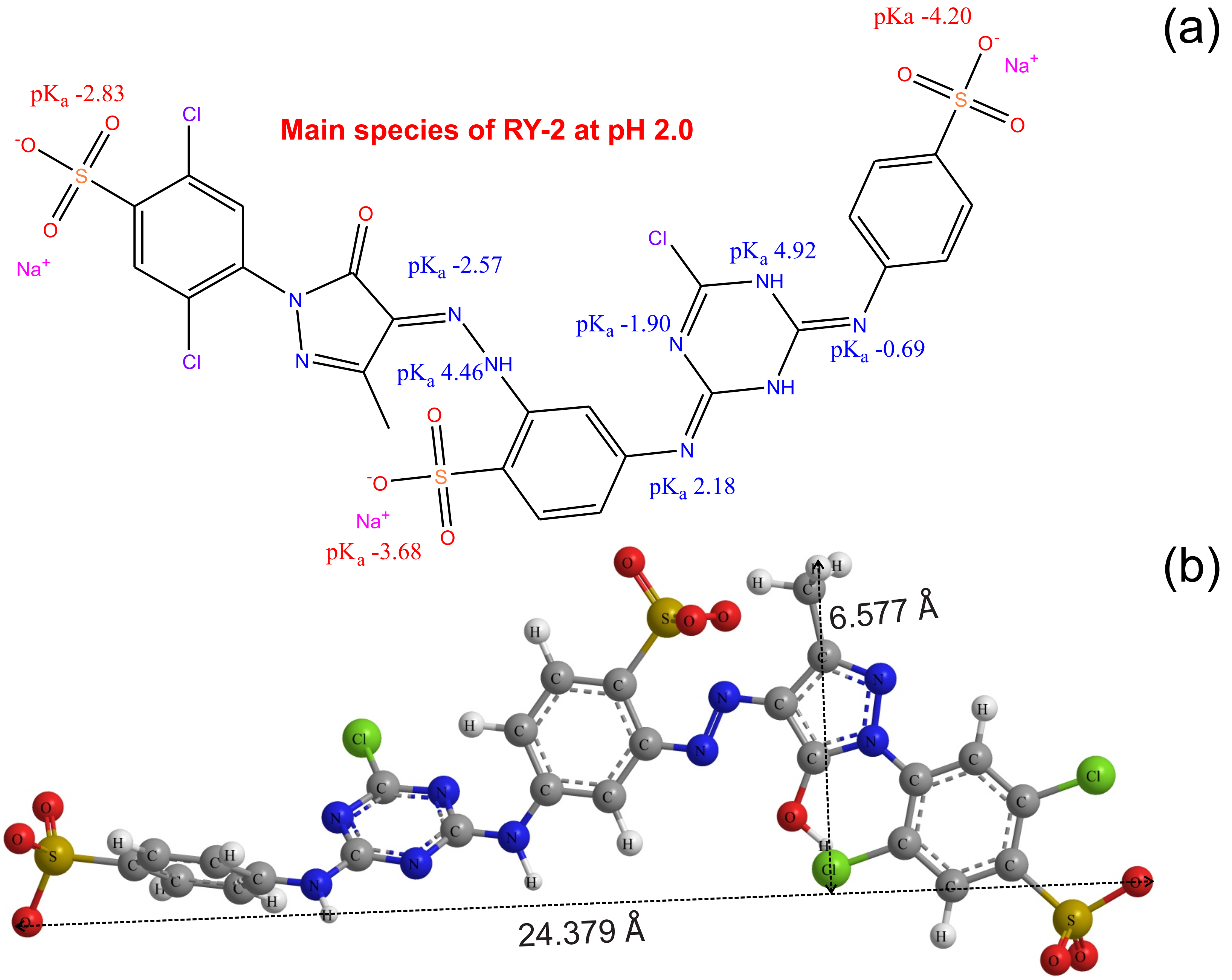

Fig S1. (a) Structural formula of Reactive Yellow 2 (RY-2; 872.947 g mol^{-1}; $C_{25}H_{15}N_9O_{10}S_3Cl_3Na_3$). pK_a values are given. (b) The optimized 3D structural formula of RY-2. The dimensions of the chemical molecule and physical-chemical properties were calculated using MarvinSketch version 24.1.2. Dipole Moment 281.79 Debye; van der Waals volume 566.32 Å^3; Polar surface area 322.24 Å²; Van der Waals surface area (3D) 865.96 Å; HLB 36.80.

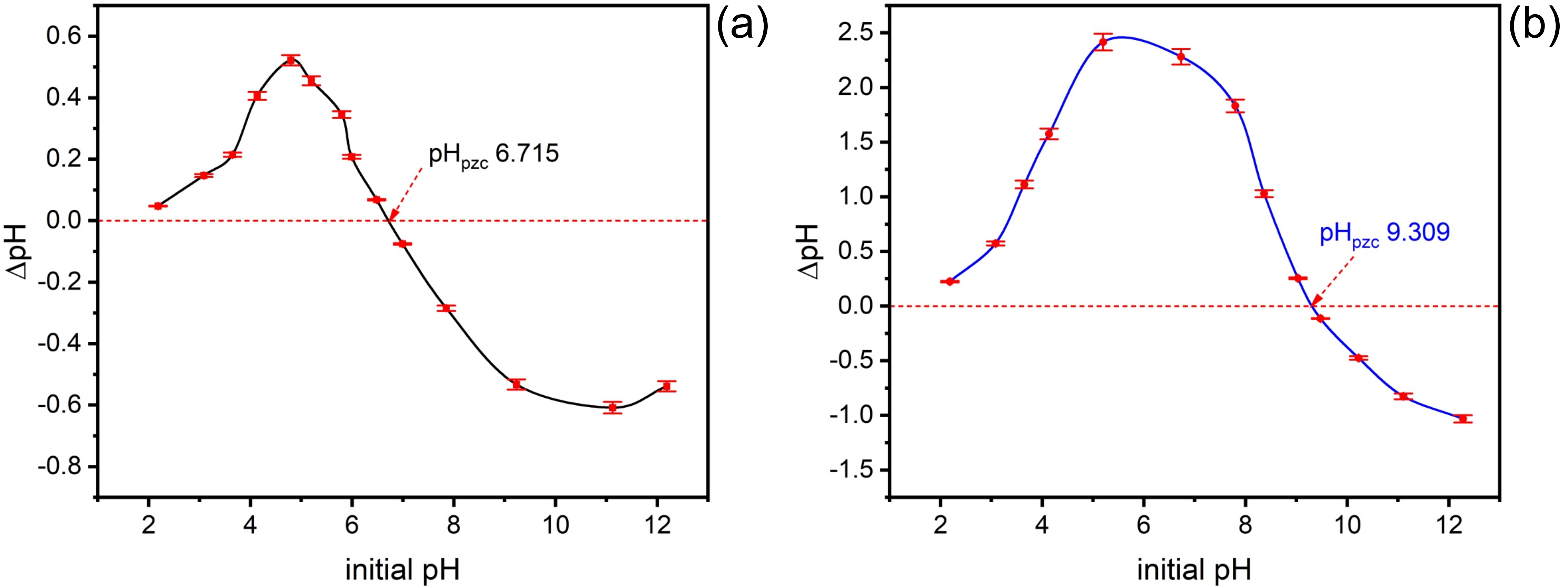

Fig S2. pH$_{pzc}$ of (a) nanocel, (b) SPEDA@nanocel.

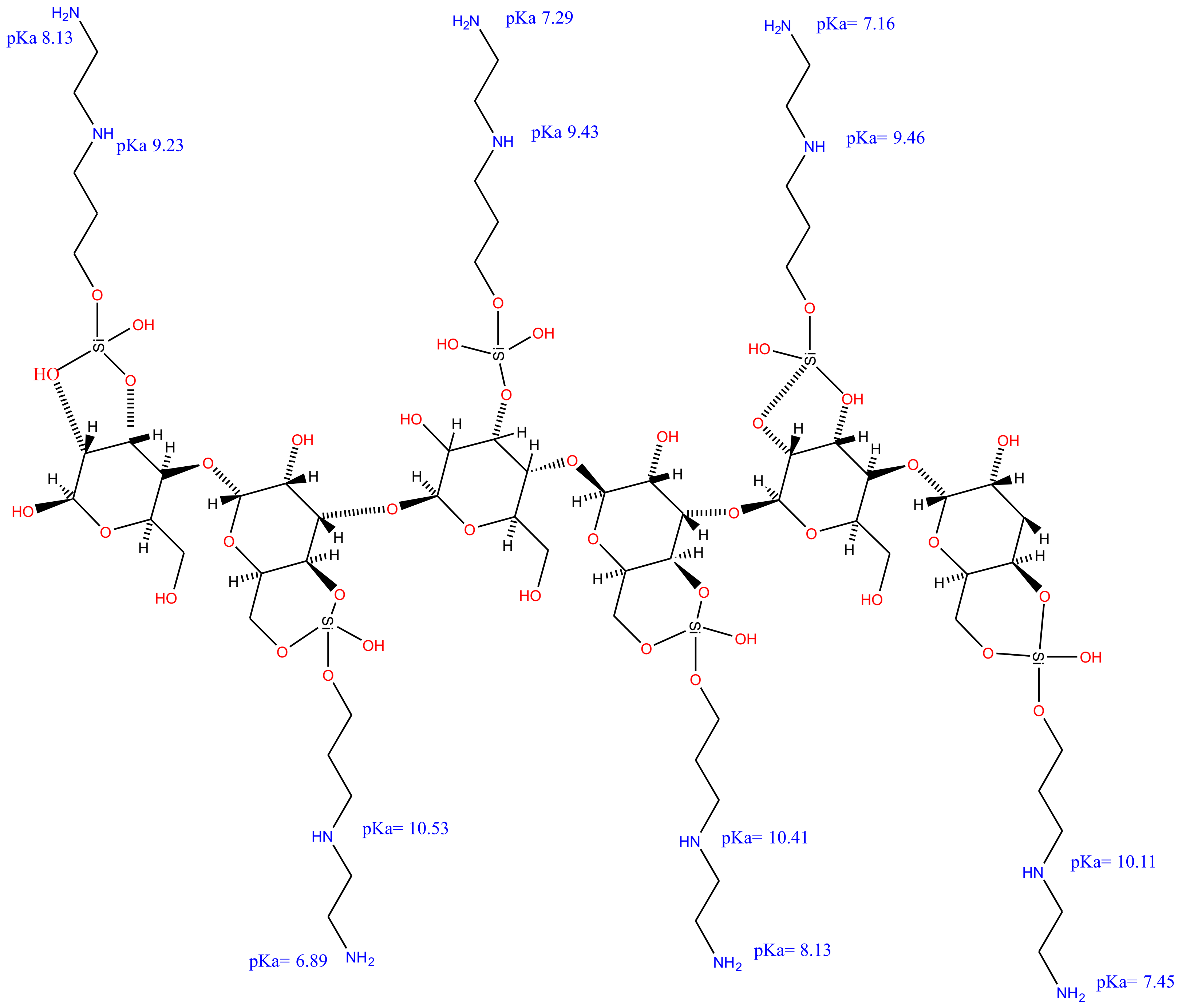

Fig S3. Estimated values of pKa of SPEDA@nanocel hybrid material and estimated isoeletric point of 9.12-9.40. Values were calculated using MarvinSketch version 24.1.2.

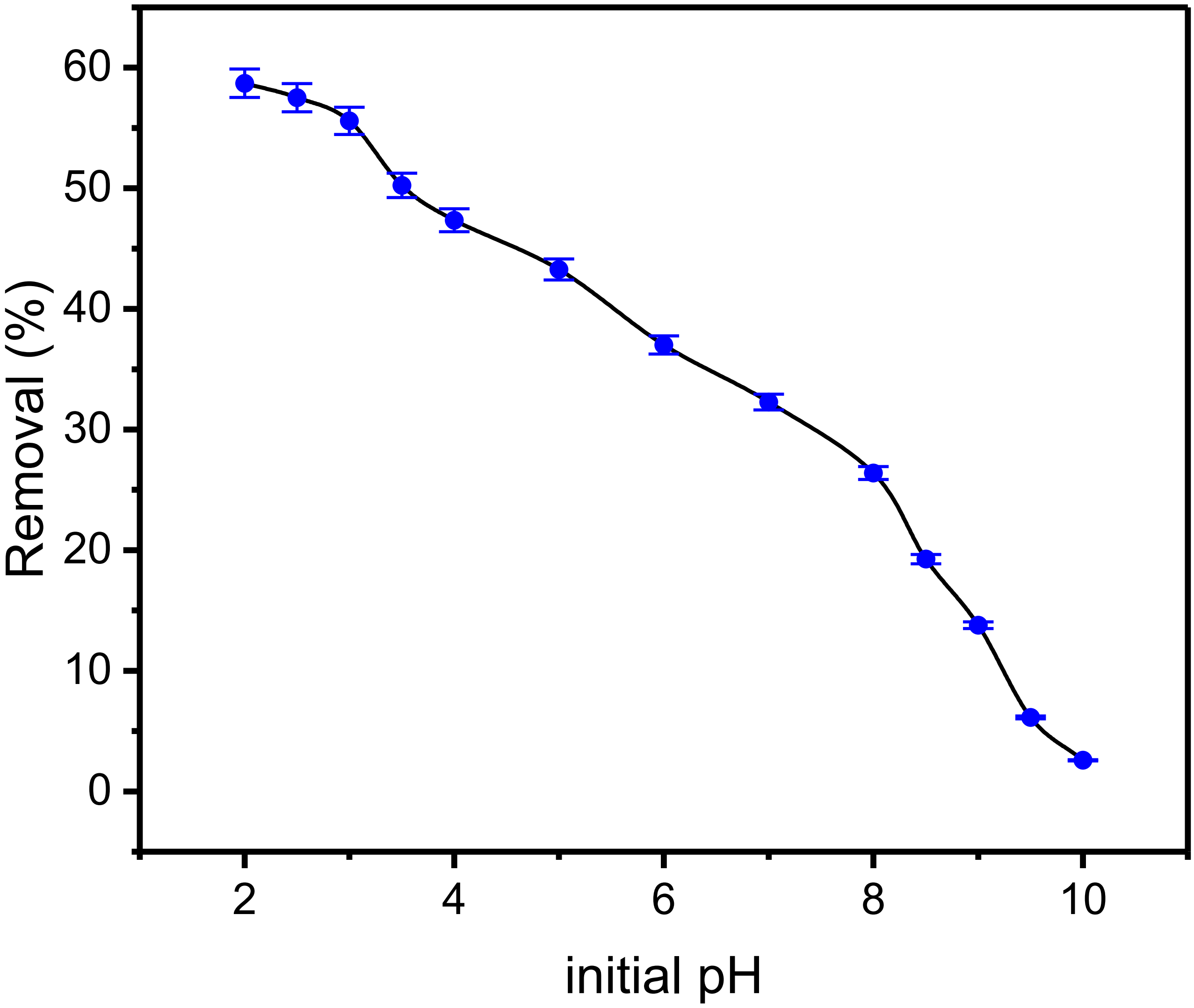

Fig S4. Effect of initial pH on the uptake of RY-2 using SPEDA@nanocel. Initial concentration 100 mg L^{-1}, adsorbent dosage 1.5 g L^{-1}, 25°C.

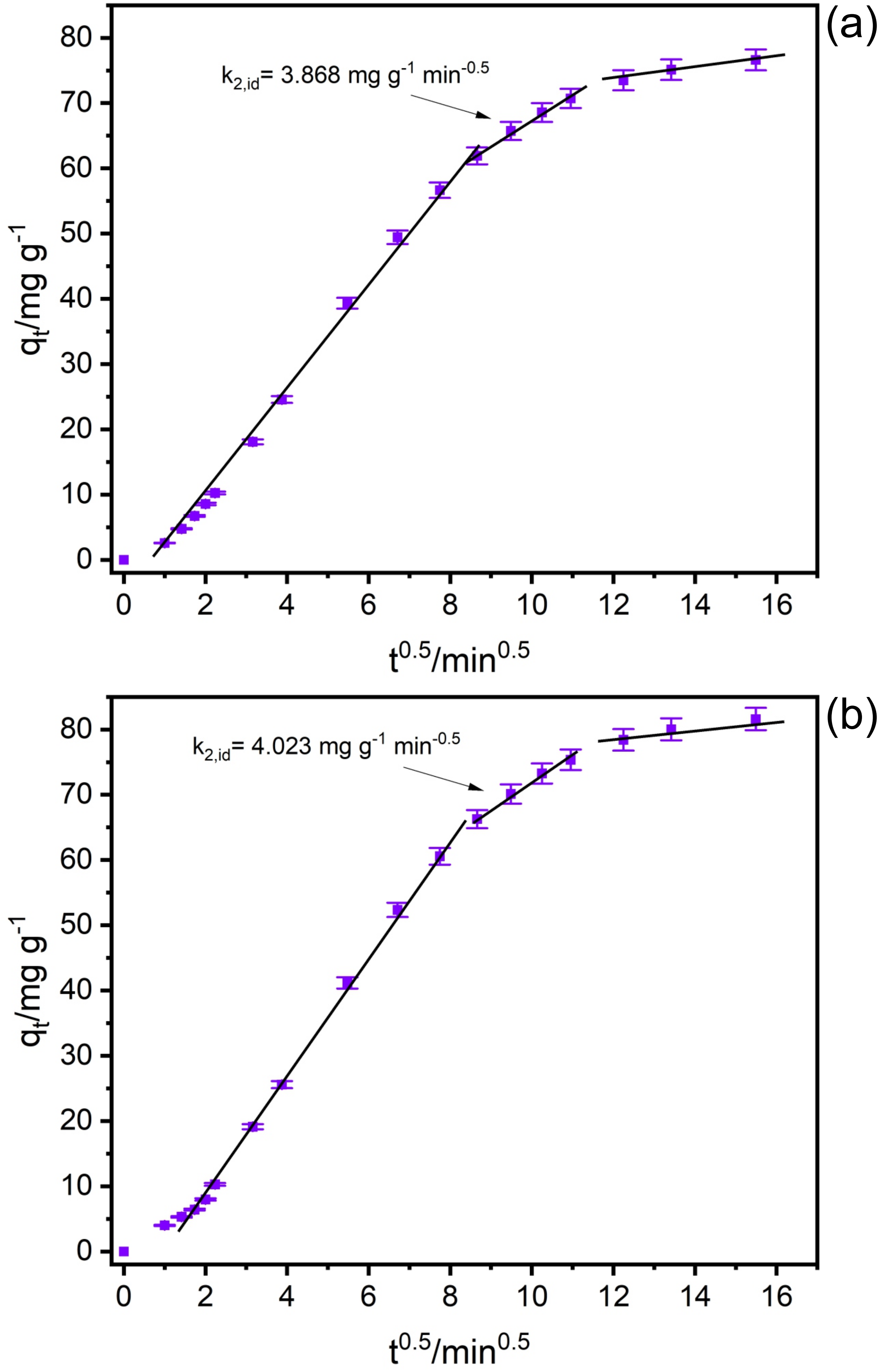

Fig S5. Intraparticle diffusion kinetics of RY-2 dye onto SPEDA@nanocel. Initial RY-2 concentration of 250 mg L^{-1} (a), and 500 mg L^{-1} (b). Temperature 25°C, adsorbent dosage 1.5 g L^{-1}, pH 2.

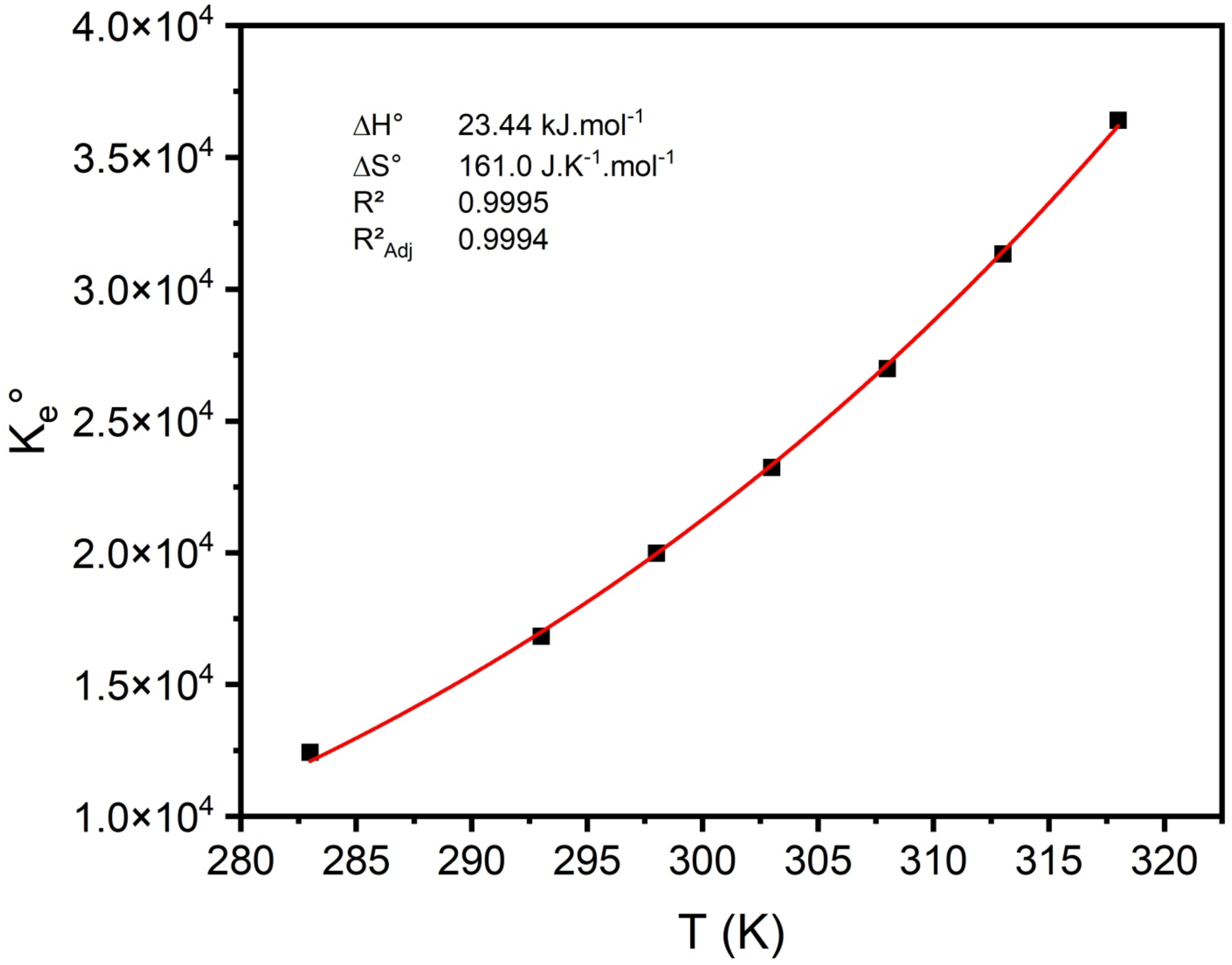

Fig S6. Nonlinear van´t Hoff curve for determination of thermodynamic adsorption parameters for the uptake of RY-2 onto SPEDA@nanocel adsorbent.
